# Can Dietary Nutrients Prevent Cancer Chemotherapy-Induced Cardiotoxicity? An Evidence Mapping of Human Studies and Animal Models

**DOI:** 10.3389/fcvm.2022.921609

**Published:** 2022-06-29

**Authors:** Xin-Yu Zhang, Ke-Lu Yang, Yang Li, Yang Zhao, Ke-Wei Jiang, Quan Wang, Xiao-Nan Liu

**Affiliations:** ^1^Ambulatory Surgery Center, Xijing Hospital, Air Force Military Medical University, Xi'an, China; ^2^Nursing Department, Chengdu BOE Hospital, Chengdu, China; ^3^Academic Center for Nursing and Midwifery, Department of Public Health and Primary Care, University of Leuven (KU Leuven), Leuven, Belgium; ^4^Department of Gastroenterological Surgery, Laboratory of Surgical Oncology, Peking University People's Hospital, Beijing, China; ^5^Department of Cardiology, Xijing Hospital, Air Force Military Medical University, Xi'an, China

**Keywords:** chemotherapy, cardiotoxicity, heart diseases, oral nutrition, diet therapy, systematic review

## Abstract

**Introduction:**

Chemotherapy has significantly improved cancer survival rates at the cost of irreversible and frequent cardiovascular toxicity. As the main dose-dependent adverse effect, cardiotoxic effects not only limit the usage of chemotherapeutic agents, but also cause the high risk of severe poor prognoses for cancer survivors. Therefore, it is of great significance to seek more effective cardioprotective strategies. Some nutrients have been reported to diminish cardiac oxidative damage associated with chemotherapy. However, the currently available evidence is unclear, which requires a rigorous summary. As such, we conducted a systematic review of all available evidence and demonstrated whether nutrients derived from food could prevent cardiotoxicity caused by chemotherapy.

**Methods:**

We searched Medline (via PubMed), Embase and the Cochrane Library from inception to Nov 9, 2021 to identify studies reporting dietary nutrients against cancer chemotherapy-related cardiotoxicity. We performed descriptive summaries on the included studies, and used forest plots to demonstrate the effects of various dietary nutrients.

**Results:**

Fifty-seven eligible studies were identified, involving 53 animal studies carried on rats or mice and four human studies in cancer patients. Seven types of dietary nutrients were recognized including polyphenols (mainly extracted from grapes, grape seeds, and tea), allicin (mainly extracted form garlic), lycopene (mainly extracted from tomatoes), polyunsaturated fatty acids, amino acids (mainly referring to glutamine), coenzyme Q10, and trace elements (mainly referring to zinc and selenium). Dietary nutrients ameliorated left ventricular dysfunctions and myocardial oxidative stress at varying degrees, which were caused by chemotherapy. The overall risk of bias of included studies was at moderate to high risk.

**Conclusion:**

The results indicated that dietary nutrients might be a potential strategy to protect cardiovascular system exposed to the chemotherapeutic agents, but more human studies are urged in this field.

**Systematic Review Registration:** https://inplasy.com/inplasy-2022-3-0015/.

## Introduction

Advances in chemotherapy and comprehensive supportive care have contributed to the steadily declined cancer mortality rates over the past decades (1–3). As a result, the survivors have been an increasingly large population (e.g., more than 16.9 million in the USA in 2019) with longer life expectancy (4, 5). However, the great success of chemotherapy has been accompanied by severe cardiovascular toxicity, which is caused by the direct damage to the myocardium through production of oxygen free radicals (5–7). Cardiac toxicity could manifest as subclinical cardiomyopathies at the early stage, such as asymptomatic changes along with left ventricular dysfunction and abnormal cardiac markers. Around 12% (123/1022) pediatric patients with acute myeloid leukemia were reported to suffer cardiotoxicity during and after the chemotherapy regimens over a five-year follow-up (8). In addition, cardiotoxicity would progress to congestive heart failure (CHF) and even cardiac death (5, 9) and these complications have been the leading cause of long-term morbidity and mortality (10–13). The incidence of CHF reported in patients treated with doxorubicin (DOX) was 2.2% (88/4018) (14) and the two-year mortality rate associated with anthracyclines-induced cardiovascular diseases (CVD) was up to 60% (15). Therefore, appropriate early prevention and management for cancer survivors should be implemented to prevent and avoid chemotherapy-induced cardiotoxic progression (16, 17).

Early detection and treatment of chemotherapy-induced cardiac damage have been gradually studied. The common used monitoring methods are echocardiography and cardiac biomarkers. Several drugs were previously investigated as cardioprotective agents for preventing cardiotoxicity (6, 7, 18, 19), but only dexrazoxane was approved by Food and Drug Administration (FDA) to protect the chemotherapy-exposed heart (7, 20). However, dexrazoxane has not been routinely applied in the clinic at present along with debate about its long-term safety. This is largely due to the concerns over its impact on anticancer treatments (21, 22). In addition, the cost and accessibility have also been quite essential impediments for cancer survivors who have already borne considerable treatment overheads in the long-term survivals (5). So, it is of great significance to explore alternative effective, safe, economical, and consistent cardiac protection strategies for long-term cancer survivors.

Dietary nutrients (defined as various nutrients derived from food) are increasingly playing an important role in medicine. Due to the restriction of conventional medicine treatments for cancer, complementary and alternative medicine (CAM) has been playing a broader and more active role in cancer patients (23). Currently, several studies indicated that some fruit and vegetables have been considered as natural antioxidants that could reduce oxidative stress and inhibit chemotherapy-related cardiotoxicity (24–26). Furthermore, dietary factors such as polyunsaturated fatty acids (PUFA) and coenzyme Q10 (CoQ10) have also been reported to be able to protect the chemotherapy-exposed heart on animal models (27, 28). Although there are some narrative reviews (27, 29–32), it seems that the evidence on whether dietary nutrients could alleviate cardiotoxicity induced by chemotherapy has not been systematically summarized.

As such, we hypothesized that dietary nutrients could serve as a novel cardioprotective strategy to prevent cancer chemotherapy-induced cardiotoxicity and conducted a systematic review of the current evidence.

## Methods

This systematic review was conducted based on the guidelines of Systematic Review Protocol for Animal Intervention Studies (33) and the Preferred Reporting Items for Systematic Reviews and Meta-Analyses (PRISMA) (34), and was registered at https://inplasy.com as INPLASY202230015.

### Inclusion and Exclusion Criteria

The inclusion criteria were as follows: (1) subjects: cancer patients or healthy/tumor-bearing animal models, treated with chemotherapeutic agents, with no restrictions on cancer types, animal species and chemotherapeutic agents; (2) intervention: oral intake of dietary nutrients; If the source of the nutrient was reported in the article, we only included cases which the nutrient source was food rather than non-food like drugs. If it was not reported, then we included articles that the nutrient can be obtained from food; (3) comparison: placebo or no intervention (without dietary nutrients mentioned above); (4) outcomes: imaging or biological measures of cardiotoxicity, including echocardiography, serum cardiac markers, oxidative stress markers, and histopathological examinations. Echocardiography is the most common and noninvasive method which measures left ventricular systolic functions like left ventricular ejection fraction (LVEF) and left ventricular fractional shortening (LVFS). It is also the most widely used screening method for monitoring cardiotoxicity both during and years after anticancer treatment (16). Cardiac markers, such as cardiac troponin (cTn), N-terminal pro-brain natriuretic peptide (NT-proBNP), creatine kinase (CK), creatine kinase-MB (CK-MB) and lactate dehydrogenase (LDH), can indicate abnormal left ventricular structure and increased cardiac stress (17). Measurements of antioxidant defense can reflect the cardiac oxidative stress status in cancer patients, including malondialdehyde (MDA), superoxide dismutase (SOD) and glutathione (GSH). The details of detection indicators are represented in Supplementary Table 1. Conference abstracts, case reports, reviews, trial protocols, duplicate publications, *in vitro* experiments, and non-controlled studies were excluded.

### Search Strategy and Study Selection

A comprehensive search was performed through three separate electronic databases, including Medline (via PubMed), Embase, and the Cochrane Library, from the inception to Nov 9, 2021. In addition, a manual search was also conducted by screening the reference lists from relevant reviews. The search strategies used are provided in Supplementary Table 2.

Two reviewers (X-YZ and K-LY) screened the titles and abstracts of records retrieved from the databases and independently screened the full text for eligible studies. Any disagreements between the two reviewers were resolved through discussion by achieving a consensus.

### Data Extraction

Two reviewers (X-YZ and K-LY) independently used a data extraction sheet to extract data from the included studies. The following information was extracted: first author, year of publication, characteristics of subjects, study design, intervention characteristics, and outcome measures. The primary outcomes included LVEF and cTn, and the secondary outcomes were LVFS, CK, CK-MB, LDH, MDA, SOD, and GSH.

### Risk of Bias Assessment

Two reviewers (X-YZ and YL) independently assessed the risk of bias for the included studies. For animal studies, we used the risk of bias tool of Systematic Review Center for Laboratory Animal Experimentation (SYRCLE). This tool is designed based on the Cochrane Risk of Bias (RoB) tool for animal experiments. It consists of 10 items, including selection bias, performance bias, detection bias, attrition bias, reporting bias, and other biases (35). Each item is rated as “Y” (low risk of bias), “N” (high risk of bias), and “U” (unclear risk of bias). For randomized controlled trials (RCT), we used the Cochrane risk of bias tool (36). It covers 6 domains of bias, namely sequence generation, allocation concealment, blinding of participants and personnel, blinding of outcome assessment, incomplete outcome data, selective outcome reporting, and other biases. The items are also assessed as “Y” (low risk of bias), “N” (high risk of bias), and “U” (unclear risk of bias). For non-randomized clinical trials and observational studies, we used the Newcastle-Ottawa Scale (NOS) which contains 8 items in three dimensions of selection, comparability, and outcome (37). It scores from 0 to 9 and higher scores show the lower risk of bias. Any disagreements were resolved by consulting a third reviewer (QW).

### Data Analysis

The primary and secondary outcomes of the review were treated as continuous variables represented by mean ± standard deviation. Number of cases and percentages were used to indicate the number of included studies. Effectiveness of dietary nutrients against cardiotoxicity was presented by the comparison between chemotherapy with dietary nutrients groups and chemotherapy groups. Statistical analyses of all outcomes were performed in forest plots using RevMan Software (Version 5.3). When there were more than two arms in the included studies, we presented all the results separately. Due to high heterogeneity from the variations in the baseline of included studies, we used a random-effects model and didn't provide a pooled result as well.

**Figure 1:**
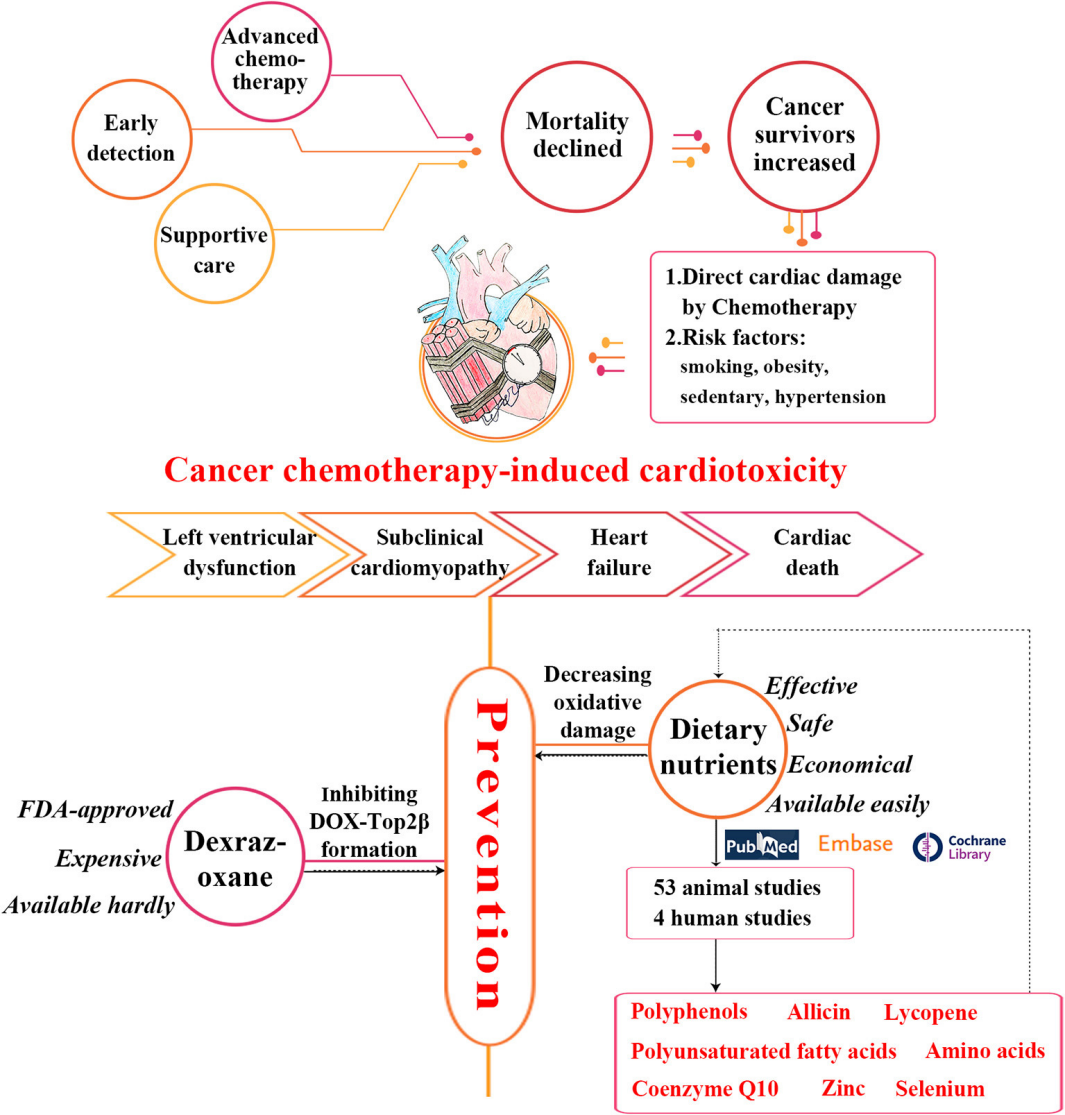
The work flowchart of the study process. Epidemiology, clinical symptoms and current cardioprotective strategies of cancer chemotherapy-induced cardiotoxicity are shown. Our search strategy and main results are also presented. DOX, doxorubicin; FDA, Food and Drug Administration; Top2β, topoisomerase 2β.

The work flowchart describing the process of the study is shown in Figure 1.

## Results

### Literature Search and Study Selection

**Figure 2:**
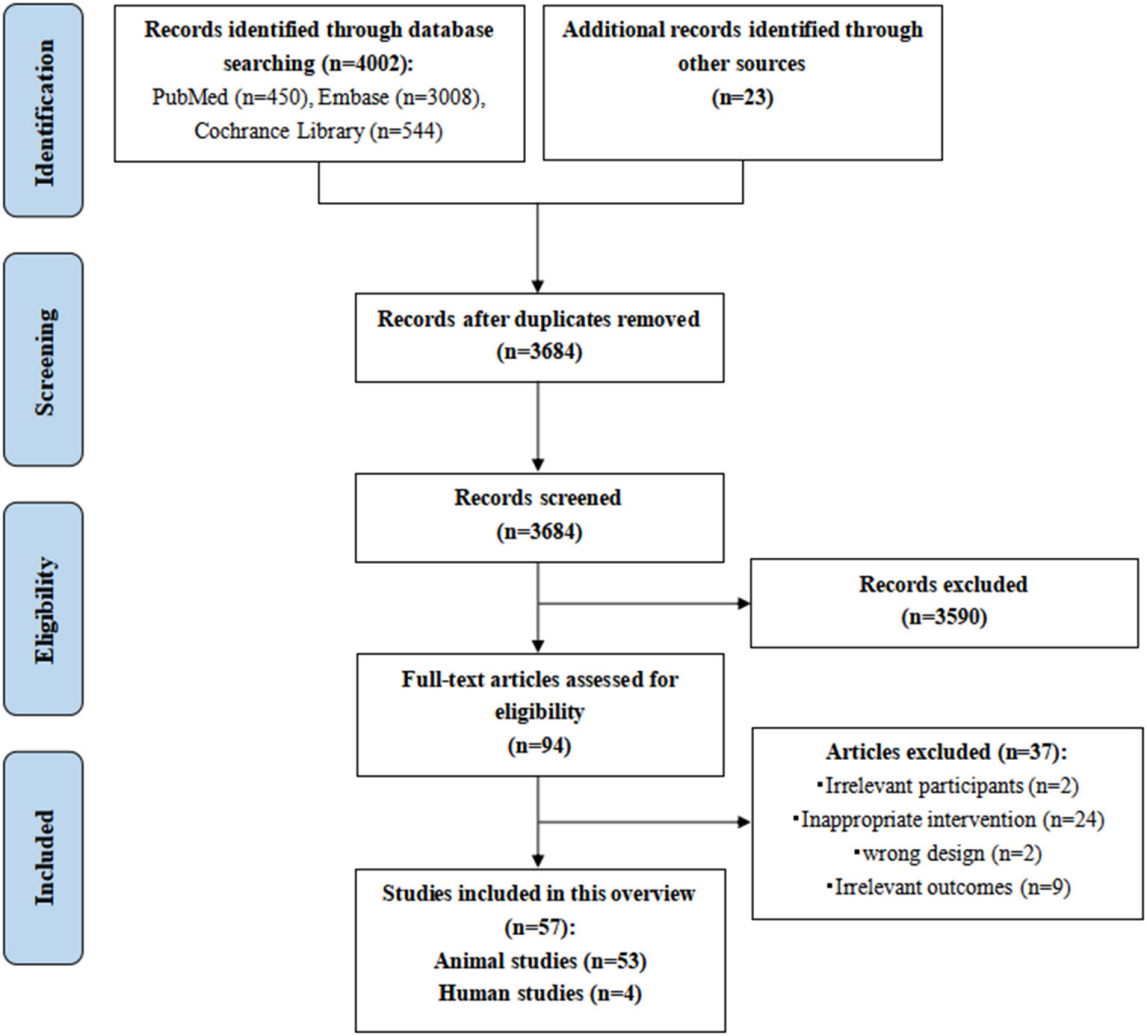
The PRISMA flowchart of the literature search and study selection.

A total of 4025 potentially relevant records were initially identified. However, 341 of those were excluded due to duplication, 3,590 studies were excluded by reading titles and abstracts based on the inclusion and exclusion criteria, and 94 potential studies were eligible for full-text screening. We finally included 57 studies, including 53 animal studies and four human studies. The PRISMA flowchart of the literature search and study selection process is shown in Figure 2. The reasons for excluding reviews are listed in Supplementary Table 3.

### Animal Studies

#### Study Characteristics

**Figure 3:**
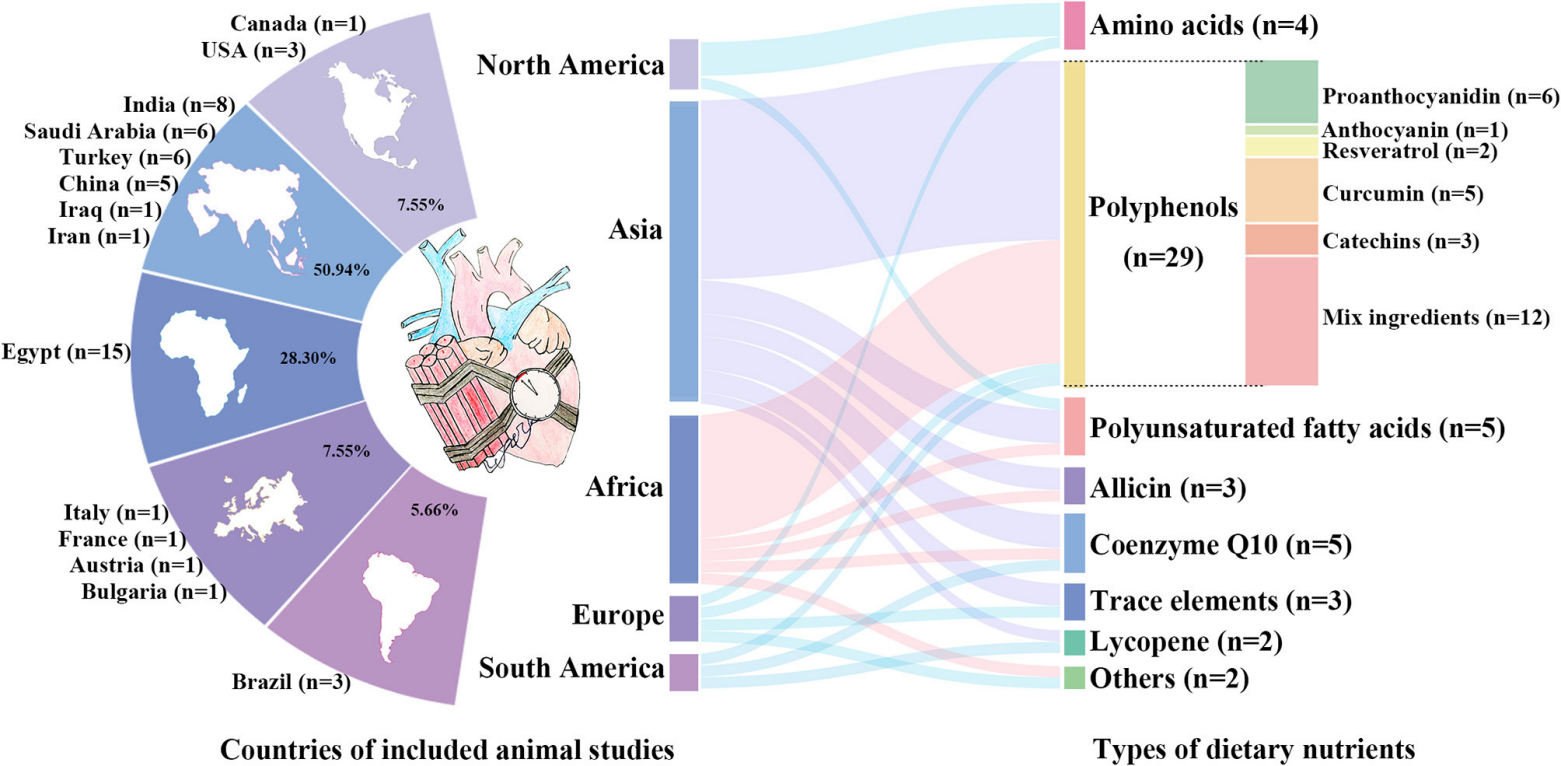
The characteristics of the included animal studies. Distribution of countries of included animal studies are shown in the left. The percentages represent the proportion of included studies in each continent, and the numbers are the number of studies in each country. Among them, African and Asian countries account for the largest share with most studies in Egypt (*n* = 15), India (*n* = 8), Saudi Arabia (*n* = 6), Turkey (*n* = 6), and China (*n* = 5). Types of dietary nutrients are listed in the right, containing polyphenols, allicin, lycopene, polyunsaturated fatty acids, amino acids, coenzyme Q10, and trace elements.

The 53 animal studies included were conducted in 14 countries, with most in Egypt (*n* = 15), India (*n* = 8), Saudi Arabia (*n* = 6), Turkey (*n* = 6), and China (*n* = 5). The publication years ranged from 1996 to 2021, with 42 before 2010. DOX (*n* = 48) comprised a significant majority of the included studies, and the other chemotherapeutic agents were cisplatin (*n* = 3), mitoxantrone (*n* = 1), and fluorouracil (*n* = 1). The covered dietary nutrients contained polyphenols (*n* = 29) (38–66), allicin (*n* = 3) (67–69), lycopene (*n* = 2) (70, 71), PUFA (*n* = 5) (72–76), amino acids (*n* = 4) (77–80), CoQ10 (*n* = 5) (81–85), trace elements (*n* = 3) (86–88), and others (*n* = 2) (89, 90). 79.24% of the included studies were investigated in Asia and Africa and most studies commonly used allicin from garlic (67–69) and polyphenols from local fruit such as grape (38–43), date palm (55, 56), cranberry (58), cardamom (59), pomegranate (60) and hawthorn (61) as the nutritional interventions. However, American and European studies tended to use amino acid like glycine and glutamine which were rich in animal food or special supplements (77–80). The characteristics of the included animal studies are summarized in Table 1 and Figure 3.

**Table 1:** The characteristics of the included animal studies.

**Dietary nutrients**	**Studies**	**Country**	**Randomization**	**Animals**			**Intervention**			**Comparison**				**Outcomes**
				**Species**	**Tumor-bearing**	**Chemotherapeutic agents**	**Food intake**	**Main ingredients**	**Duration**	**Sample size**	**Grouping**	**Control groups**	**Treated groups**	
Polyphenols	Adiyaman et al. (38)	Turkey	Not reported	Rats, sprague dawley	Healthy	DOX	Grape seed extract	Proanthocyanidin	35 days	28	4	(1) CON (2) grape seed extract (3) DOX	(4) DOX+grape seed extract	b, c, d
	Ammar et al. (39)	Egypt	Not reported	Rats, sprague dawley	Healthy	DOX	Proanthocyanidin	Proanthocyanidin	10 days	24	4	(1) CON (2) proanthocyanidin (3) DOX	(4) DOX+proanthocyanidin	b, c, e
	Boghdady (40)	Egypt	Yes	Rats, wistar albino	Healthy	DOX	Grape seed extract	Proanthocyanidin	15 days	32	4	(1) CON (2) DOX	(3) DOX+grape seed extract (4) DOX+ginkgo biloba extract	b, c, d
	Yalcin et al. (41)	Turkey	Yes	Mice, albino	Healthy	DOX	Grape seed extract	Proanthocyanidin	21 days	36	6	(1) CON (2) DOX (3) grape seed extract 50 (4) grape seed extract 150	(5) DOX+grape seed extract 50 (6) DOX+grape seed extract 150	c, d
	Yousef et al. (42)	Egypt	Not reported	Rats, sprague dawley	Healthy	Cisplatin	Grape seed extract	Proanthocyanidin	15 days	32	4	(1) CON (2) grape seed extract (3) cisplatin	(4) cisplatin+grape seed extract	b, c, f
	Zhang et al. (43)	China	Yes	Mice, balb/c	Sarcoma	DOX	Proanthocyanidin	Proanthocyanidin	10 days	56	4	(1) CON (2) DOX (3) proanthocyanidin	(4) DOX+proanthocyanidin	b, c
	Petroni et al. (44)	Italy	Not reported	Mice, c57bl/6j	Healthy	DOX	Cyanidin 3-glucoside	Anthocyanin	74 days	24	2	(1) DOX+yellow diet	(2) DOX+red diet	f
	Shoukry et al. (45)	Egypt	Yes	Rats, wister	Healthy	DOX	Resveratrol	Resveratrol	42 days	32	4	(1) CON (2) DOX	(3) DOX+resveratrol(pre) (4) DOX+resveratrol(post)	a, b, d, f
	Arafa et al. (46)	Egypt	Not reported	Rats, wistar albino	Healthy	DOX	Resveratrol	Resveratrol	28 days	40	4	(1) CON (2) resveratrol (3) DOX	(4) DOX+resveratrol	b, c, d, f
	Ibrahim Fouad and Ahmed. (47)	Egypt	Yes	Rats, wistar albino	Healthy	DOX	Curcumin	Curcumin	/	24	4	(1) CON (2) DOX (3) curcumin	(4) DOX+curcumin	b, c
	Bahadir et al. (48)	Turkey	Yes	Rats, wistar albino	Healthy	Cisplatin	Curcumin	Curcumin	14 days	49	7	(1) CON (2) placebo (3) cisplatin (4) beta-carotene (6) curcumin	(5) cisplatin+beta-carotene (7) cisplatin+curcumin	b, c, d
	Benzer et al. (49)	Turkey	Yes	Rats, wistar albino	Healthy	DOX	Curcumin	Curcumin	7 days	35	5	(1) CON (2) curcumin 200 (3) DOX	(4) DOX+curcumin 100 (5) DOX+curcumin 200	b, c, d
	Swamy et al. (50)	India	Yes	Rats, albino	Healthy	DOX	Curcumin	Curcumin	14 days	24	4	(1) CON (2) DOX (3) curcumin	(4) DOX+curcumin	b, c, d, f
	Venkatesan (51)	India	Not reported	Rats, wistar	Healthy	DOX	Curcumin	Curcumin	7 days	24	4	(1) CON (2) curcumin (3) DOX	(4) DOX+curcumin	b, c, e
	Ibrahim et al. (52)	Saudi Arabia	Yes	Mice, balb/c	Healthy	Cisplatin	Green tea extract, vitamin E	Catechins, vitamin E	30 days	48	6	(1) CON (2) green tea extract (3) vitamin E (4) cisplatin	(5) cisplatin+green tea extract (6) cisplatin+vitamin E	b, c, f
	Saeed et al. (53)	Egypt	Yes	Rats, wistar	Healthy	DOX	Epigallocatechin-3-gallate	Catechins	12 days	40	5	(1) CON (2) DOX	(3) DOX+epigallocatechin-3-gallate 10 (4) DOX+epigallocatechin-3-gallate 20 (5) DOX+epigallocatechin-3-gallate 40	b, c, d, e
	Amanullah et al. (54)	India	Not reported	Rats, wistar albino	Healthy	DOX	Black tea extract, resveratrol	Catechins, polyphenols	30 days	30	6	(1) CON (2) DOX (3) black tea extract+ resveratrol	(4) DOX+black tea extract (5) DOX+resveratrol (6) DOX+black tea extract +resveratrol	b, c, f
	Mubarak et al. (55)	Egypt	Not reported	Rats, albino	Healthy	DOX	Date palm fruit extract	Anthocyanins, quercetin, procyanidins	30 days	40	4	(1) CON (2) date (3) DOX	(4) DOX+date	b, c
	Sabbah et al. (56)	Saudi Arabia	Yes	Rats, wistar albino	Healthy	DOX	Ajwa date aqueous extract	Polyphenols, flavonoids, Mn	28 days	60	6	(1) CON (2) date 0.75 (3) date 1.5 (4) DOX	(5) DOX+date 0.75 (6) DOX+date 1.5	b, c, d
	Ribeiro et al. (57)	Brazil	Not reported	Rats, wistar	Healthy	DOX	Pera orange juice, Moro orange juice	Hesperidin, anthocyanins	28 days	120	6	(1) CON (2) Pera juice (3) Moro juice (4) DOX	(5) DOX+Pera juice (6) DOX+Moro juice	a, c, f
	Elberry et al. (58)	Saudi Arabia	Yes	Rats, wister	Healthy	DOX	Cranberry extract	Flavonols, flavonoids	10 days	30	4	(1) CON (2) cranberry extract (4) DOX	(4) DOX+cranberry extract	b, c, e
	Abu Gazia and El-Magd (59)	Egypt	Yes	Rats, albino	Healthy	DOX	Cardamom extract	Flavonoids	21 days	30	3	(1) CON (2) DOX	(3) DOX+cardamom extract	b, c, d
	Hassanpour Fard et al. (60)	India	Not reported	Rats, wistar albino	Healthy	DOX	Whole fruit extract of pomegranate	Gallic acid, quercetin	18 days	24	3	(1) CON (2) DOX	(3) DOX+pomegranate extract	b, c, d, e, f
	Shatoor and Said Ahmed, (61)	Saudi Arabia	Yes	Rats, wistar albino	Healthy	DOX	Hawthorn extrat	Flavonoids, polyphenols	28 days	36	6	(1) CON (2) hawthorn (3) DOX	(4) DOX+hawthorn(st) (5) DOX+hawthorn(post) (6) hawthorn+DOX(pre)	b, c, d, f
	Subburaman et al. (62)	India	Not reported	Rats, albino	Healthy	DOX	Naringenin	Flavonoids	70 days	18	3	(1) CON (2) DOX	(3) DOX+naringenin	b, c, d, f
	Abdel-Wahab et al. (63)	Egypt	Not reported	Rats, swiss albino	Healthy	DOX	P-coumaric acid	p-coumaric acid (pca)	5 days	24	4	(1) CON (2) P-coumaric acid (3) DOX	(4) DOX+p-coumaric acid	b, c
	Alhumaydhi (64)	Saudi Arabia	Yes	Mice, balb/c	Healthy	DOX	Honey	Polyphenols, fructose, glucose	10 days	40	4	(1) CON (2) honey (3) DOX	(4) DOX+honey	b
	Abu-Elsaad et al. (65)	Egypt	Not reported	Rats, sprague dawley	Healthy	DOX	Tested food: yogurt, green tea extract, carrot	Lactobacillus acidophilus, polyphenols, carrot	154 days	60	5	(1) CON (2) DOX (3) DOX+carvedilol	(4) DOX+tested food (5) DOX+tested food+carvedilol	b, c, d, e
	Lin et al. (66)	China	Yes	Rats, sprague dawley	Healthy	DOX	Yellow wine polyphenolic compounds	Polyphenolic compounds	28 days	50	5	(1) CON (2) yellow wine (3) DOX	(4) DOX+yellow wine	a, c, d, e, f
Allicin	Abdel-Daim et al. (67)	Egypt	Yes	Mice, swiss albino	Healthy	DOX	Allicin	Allicin	14 days	40	5	(1) CON (2) allicin 20 (3) DOX	(4) DOX+allicin 10 (5) DOX+allicin 20	b, c
	Demirkaya et al. (68)	Turkey	Yes	Rats,wistar albino	Healthy	DOX	Aged garlic extract, grape seed extract, hazelnut	Allicin, proanthocyanidin	42 days	135	9	(1) CON (2) DOX 15 (3) DOX 7.5	(4) DOX 15+aged garlic extract (5) DOX 7.5+aged garlic extract (6) DOX 15+grape seed extract (7) DOX 7.5+grape seed extract (8) DOX 15+ hazelnut (9) DOX 7.5+hazelnut	b, c, d
	Mukherjee et al. (69)	India	Not reported	Rats, wistar albino	Healthy	DOX	Garlic homogenate	Allicin	30 days	40	5	(1) CON (2) DOX (3) DOX+PRO	(4) DOX+garlic 250 (5) DOX+garlic 500	c, f
Lycopene	Ferreira et al. (70)	Brazil	Not reported	Rats, wistar	Healthy	DOX	Tomato-oleoresin supplement	Lycopene	49 days	34	4	(1) CON (2) lycopene (3) DOX	(4) DOX+lycopene	d
	Yilmaz et al. (71)	Turkey	Not reported	Rats, sprague dawley	Healthy	DOX	Lycopene	Lycopene	10 days	24	4	(1) CON (2) DOX	(3) DOX+lycopene(pre) (4) DOX+lycopene(post)	c, d
PUFA	Ahmed et al. (72)	India	Yes	Rats, wistar	Healthy	DOX	Chia seed oil	PUFA	7 days	24	4	(1) CON (2) DOX	(3) DOX+chia seed oil 2.5 (4) DOX+chia seed oil 5	b, c, d, e
	Asselin et al. (73)	Canada	Yes	Mice, c57bl/6	Healthy	DOX+TRZ	Flaxseed, α-linolenic acid, secoisolariciresinol diglucoside	α-Linolenic acid, secoisolariciresinol diglucoside	42 days	84	5	(1) CON (2) DOX+TRZ	(3) DOX+TRZ+flaxseed (4) DOX+TRZ+α-linolenic acid (5) DOX+TRZ+secoisolariciresinol diglucoside	a, d, e
	Saleh et al. (74)	Egypt	Not reported	Rats, wistar albino	Healthy	DOX	N-3 PUFA	n-3 PUFA	28 days	35-40	5	(1) CON (2) DOX	(3) DOX+n-3 PUFA 25 (4) DOX+n-3 PUFA 50 (5) DOX+n-3 PUFA100	b, c, d, e, f
	Saleem et al. (75)	India	Not reported	Rats, wistar albino	Healthy	DOX	Sesame oil	Linoleic acid, α-linolenic acid, sesamin	30 days	30	5	(1) CON (2) DOX (5) DOX+probucol	(3) DOX+sesame oil 1 (4) DOX+sesame oil 2	b, c, d
	Teng et al. (76)	China	Yes	Rats, sprague dawley	Healthy	DOX	N-3 PUFA	Timnodonic acid, docosahexaenoic acid	112 days	32	3	(1) CON (2) DOX	(3) DOX+n-3 PUFA	a, d
Amino acids	Maneikyte et al. (77)	Austria	Yes	Rats, wag/rij	Colorectal cancer liver metastasis	FOLFOX	Glycine	Glycine	21 days	44	6	(1) casein+sham (2) glycine+sham (3) casein+CON (5) casein+FOLFOX	(4) glycine+CON (6) glycine+FOLFOX	a, b, d
	Todorova et al. (78)	USA	Yes	Rats, fisher344	Mammary carcinoma	DOX	Glutamine	Glutamine	/	50	3	(1) CON (2) DOX+water	(3) DOX+glutamine	a, b
	Todorova et al. (79)	USA	Yes	Rats, fisher344	Mammary carcinoma	DOX	Glutamine	Glutamine	7 days	20	2	(1) DOX+CON	(2) DOX+glutamine	a, c
	Cao et al. (80)	USA	Yes	Rats, fisher 344	Healthy	DOX	Glutamine	Glutamine	28 days	42	6	(1) H_2_O+saline (2) H_2_O+DOX	(3) glutamine+saline (4) glutamine+DOX	c
CoQ10	Rahmanifard et al. (81)	Iran	Yes	Rats, sprague dawley	Healthy	DOX	CoQ10	CoQ10	21 days	42	6	(1) CON (2) lisinopril (3) CoQ10 (4) DOX	(5) DOX+lisinopril (6) DOX+CoQ10	c, d, e, f
	Shabaan et al. (82)	Egypt	Yes	Rats, wistar	Healthy	DOX	CoQ10	CoQ10	7 days	28	4	(1) CON (2) CoQ10 (3) DOX	(4) DOX+CoQ10	c, d
	Botelho et al. (83)	Brazil	Yes	Rats, wistar albino	Healthy	DOX	CoQ10	CoQ10	14 days	20	4	(1) CON (2) CoQ10 (3) DOX	(4) DOX+CoQ10	b, c, d, e
	Chen et al. (84)	China	Yes	Rats, sprague dawley	Healthy	DOX	CoQ10	CoQ10	21 days	24	4	(1) CON (2) DOX (4) CoQ10	(3) DOX+CoQ10	d, f
	Mustafa et al. (85)	Saudi Arabia	Not reported	Rats, wistar albino	Healthy	DOX	CoQ10	CoQ10	15 days	72	6	(1) CON (2) DOX (3) CoQ10	(4) DOX+CoQ10	b, c, e, f
Trace elements	Maryoosh et al. (86)	Iraq	Yes	Rats, wistar albino	Healthy	mitoxantrone	Zinc sulfate	Zinc	20 days	48	6	(1) CON (2) Zinc 15 (3) Zinc 30 (4) mitoxantrone	(5) mitoxantrone+Zinc 15 (6) mitoxantrone+Zinc 30	b, c
	Wu et al. (87)	China	Yes	Rats, sprague dawley	Healthy	DOX	ZnCM	Zinc, curcumin	28 days	42	6	(1) CON (2) DOX	(3) DOX+curcumin 100 (4) DOX+ZnCM 25 (5) DOX+ZnCM 50 (6) DOX+ZnCM 100	a, b, e
	Coudray et al. (88)	France	Not reported	Rats, wistar	Healthy	DOX	Selenium	Selenium	49 days	60	5	(1) CON (2) saline (3) DOX (4) selenium	(5) DOX+selenium	c, f
Others	Radeva-Ilieva et al. (89)	Bulgaria	Yes	Rats, wistar	Healthy	DOX	Methylxanthine from bancha	Methylxanthine	17 days	36	6	(1) CON (2) DOX (3) methylxanthine 5 (4) methylxanthine 1	(5) DOX+methylxanthine 5 (6) DOX+methylxanthine 1	b
	Wahab et al. (90)	Egypt	Yes	Mice, swiss albino	Ehrlich ascites carcinoma	DOX	Vitamin E	Vitamin E	30 days	140	4	(1) DOX	(2) DOX+vitamin E	c

*CON, control; CoQ10, coenzyme Q10; DOX, doxorubicin; FOLFOX, 5-fluorouracil+leucovorin+oxaliplatin; PUFA, polyunsaturated fatty acids; TRZ, trastuzumab; ZnCM, zinc+curcumin*.

*a, echocardiography; b, serum cardiac markers; c, oxidative stress markers; d, histopathological examinations; e, electrocardiogram; f, survival, body weight, heart weight*.

#### Risk of Bias Assessment

**Figure 4:**
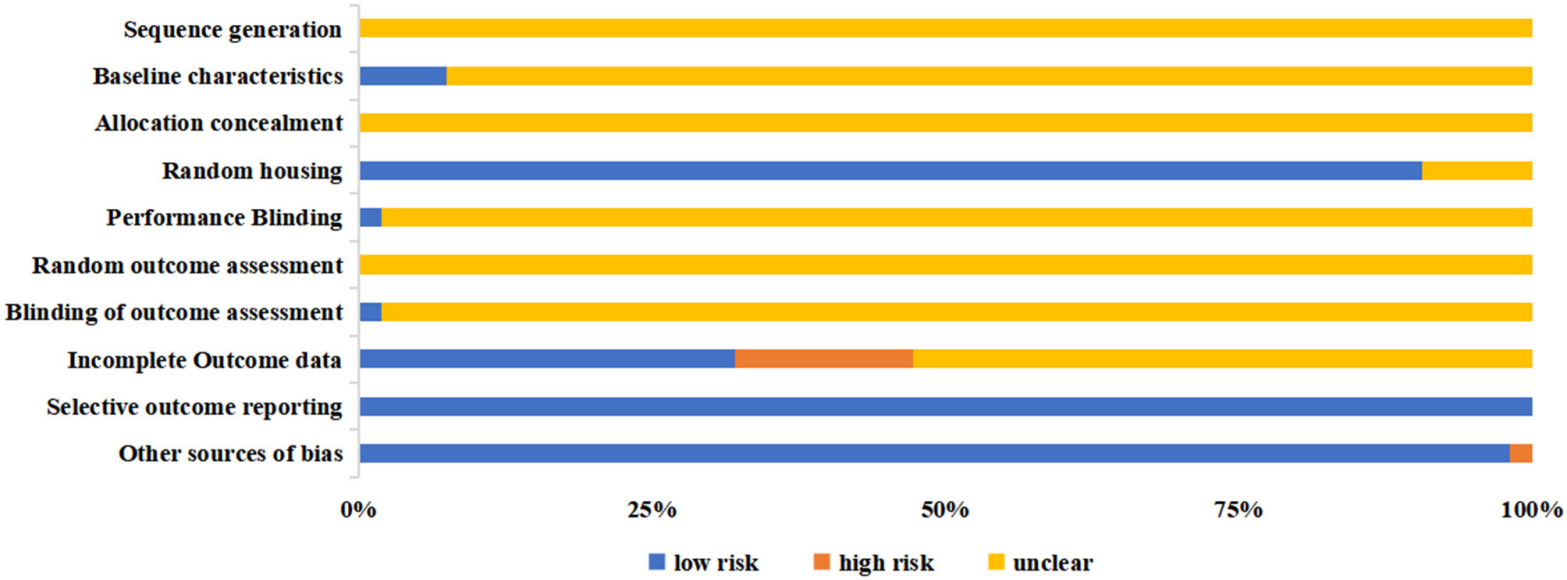
The risk of bias assessment of the included animal studies.

The overall risk of bias of included animal studies was at moderate to high risk and most of the items of SYRCLE depicted unclear risk (Figure 4 and Supplementary Table 4). All animal studies failed to report the sequence generation methods (item 1), allocation concealment (item 3), and random outcome assessment (item 6). Other items also revealed poor outcomes. Four studies (73, 77–79) reported baseline characteristics (item 2), one (84) mentioned the methods of performance blinding (item 5), and another (77) documented the blinded outcome assessment (item 7). In comparison, all the animal studies were free from selective outcome reporting (item 9), and 52 studies did not involve any other sources of bias (item 10).

#### Effectiveness of Dietary Nutrients

The protective effects of dietary nutrients above on myocardium against cardiac toxicity can be observed by the comparison between chemotherapy with nutrients groups and chemotherapy groups in Supplementary Figures 1–7.

##### Polyphenols

In animal studies of our review, polyphenols were defined as a broad class of compounds with multiple phenolic hydroxyls (PhOH) and were reported in 29 studies (38–66). These covered proanthocyanidin (*n* = 6, all derived from grape seed extract) (38–43), anthocyanin (*n* = 1, derived from purple corn) (44), resveratrol (*n* = 2) (45, 46), curcumin (*n* = 5) (47–51), catechins (*n* = 3, derived from green tea and black tea) (52–54), and mixed phenolic compounds extracted from local products [*n* = 12, derived from date (55, 56), orange (57), cranberry (58), cardamom (59), pomegranate (60), hawthorn (61), naringenin (62), p-coumaric acid (63), honey (64), yogurt (65) and yellow wine (66)]. Three studies demonstrated that supplementation of polyphenols could improve DOX-induced cardiac dysfunctions evaluated by echocardiography (45, 57, 66). LVEF significantly reduced in DOX groups, but was similar in DOX+Nutrients groups and control groups. Cardiac morphological and systolic changes led by DOX were attenuated by polyphenolic nutrients through scavenging free radicals and blocking lipid peroxidation. Biochemical analyses were estimated by serum cardiac markers and antioxidant parameters in 28 studies (38–43, 45–66). LDH, MDA and SOD were reported most. The concentrations of myocardial enzymes in animals received chemotherapy and nutrients were significantly lower compared with those treated with chemotherapeutic agents alone. Oral administration of polyphenols improved the cardiac oxidative changes led by chemotherapy and enhanced the antioxidant enzymatic activities. Histopathological analyses of cardiac tissue captured under the microscope were reported in 16 studies (38, 40, 41, 45, 46, 48–50, 53, 56, 59–62, 65, 66). The incidences of myocardial atrophy, cytoplasmic vacuoles, nuclear pyknosis, and cytoplasmic eosinophilia were significantly higher in heart exposed to DOX, while the polyphenolic substance protected or even restored cardiac disrupted histological structure induced by DOX (Supplementary Figure 1).

##### Allicin and Lycopene

Three studies showed that allicin (all derived from garlic extract) effectively decreased the expression of myocardial tumor necrosis factor-alpha (TNF-α) and mitigated cardiac oxidative damage (67–69) (Supplementary Figure 2). Abdel-Daim et al. (67) referred that allicin could be a promising cytoprotective agent against DOX-related cardiotoxicity. Two studies revealed that lycopene (all derived from tomatoes) reduced the levels of cardiac oxidative markers and made the histopathological changes maintain nearly normal after the injection of DOX (70, 71) (Supplementary Figure 3).

##### Polyunsaturated Fatty Acids

PUFA were reported in five studies, derived from black chia seed (72), flaxseed (73), fish oil (74, 76), and sesame oil (75). All these studies proved that PUFA attenuated the myocardial necrosis and overall myocardium enlargement and alleviated histopathological alteration in rats/mice treated with DOX (Supplementary Figure 4). PUFA were considered as a potential chemoprotectant nutraceutical in combination with chemotherapy to limit the cardiotoxic side effects (72).

##### Amino Acids, Coenzyme Q10, and Trace Elements

Four studies reported that amino acids [derived from glycine (77) and glutamine (78–80)] could diminish chemotherapy-induced cardiac oxidative damage (Supplementary Figure 5). As a vital role in maintaining the cellular redox state, dietary glutamine remained normal cardiac GSH levels in animal models treated with chemotherapeutic drugs and prevented cardiac lipid peroxidation (78–80). Coenzyme Q10 (*n* = 5) (81–85) was proven to be prophylactic in prevention of cardiovascular toxicity through participating with redox function directly in the mitochondrial respiratory chain (Supplementary Figure 6), and trace elements [*n* = 3, derived from zinc (Zn) (86, 87) and selenium (Se) (88)] were also exhibited to protect myocardium by preventing mitochondrial dysfunctions and acting in concert with SOD and catalase (Supplementary Figure 7).

The details of outcomes are summarized in Supplementary Table 5.

### Human Studies

#### Study Characteristics

Four human studies were conducted in Egypt [in 2021 (91) and 2020 (92)], Italy [in 1994 (93)] and the USA [in 1978 (94)]. Three studies (91–93) recruited pediatric patients diagnosed with acute lymphoblastic leukemia (ALL) aged 1 to 16 years and one (94) recruited adults with bronchogenic carcinoma. Two studies (91, 92) were RCTs using DOX as the chemotherapy agent, and the other two studies (93, 94) were non-randomized controlled trials using anthracyclines. The covered dietary nutrients contained PUFA [*n* = 2, derived from omega 3 fatty acids (91) and black seed oil (92)] and CoQ10 (*n* = 2) (93, 94). The characteristics of the included human studies are summarized in Table 2.

**Table 2:** The characteristics of the included human studies.

**Dietary nutrients**	**Studies**	**Country**	**Study design**	**Participants**				**Intervention**			**Comparison**				**Outcomes**
				**Cancer type**	**Gender**	**Age**	**Chemotherapeutic agents**	**Food intake**	**Main ingredients**	**Duration**	**Sample size**	**Grouping**	**Control groups**	**Treated groups**	
PUFA	El Amrousy et al. (91)	Egypt	RCT	Acute lymphoblastic leukemia	Male, 36 Female, 24	8.7 ± 1.9 years old	DOX	Omega 3 fatty acids	Omega 3 fatty acids	180 days	60	2	(1) DOX	(2) DOX+omega 3 fatty acids	a, b, c
	Hagag et al. (92)	Egypt	RCT	Acute lymphoblastic leukemia	Male, 25 Female, 15	2-16 years old	DOX	Black seed oil	PUFA (linoleic acid, oleic acid, palmitic acid)	7 days	40	2	(1) DOX+placebo	(2) DOX+black seed oil	a
CoQ10	Iarussi et al. (93)	Italy	Non-randomized controlled trial	Acute lymphoblastic leukemia, non-hodgkin lymphoma	/	1-15 years old	Anthracyclines	CoQ10	CoQ10	/	20	2	(1) anthracyclines	(2) anthracyclines+CoQ10	a
	Cortes et al. (94)	USA	Non-randomized controlled trial	Bronchogenic carcinoma, other carcinoma	Male, 11 Female, 7	56.87 years old	DOX	CoQ10	CoQ10	150 days	18	2	(1) DOX	(2) DOX+CoQ10	a, d

*CON, control; CoQ10, coenzyme Q10; DOX, doxorubicin; PUFA, polyunsaturated fatty acids; RCT, randomized controlled trial*.

*a: echocardiography; b: serum cardiac markers; c: oxidative stress markers; d: electrocardiograph*.

#### Risk of Bias Assessment

The overall risk of bias of included human studies was at moderate to high risk. All items of Cochrane risk of bias tool were rated as low risk in one RCT (91), and three items (blinding of participants and personnel, blinding of outcome assessment, and selective outcome reporting) were rated as unclear risk in another RCT (92). The NOS's scores of the non-randomized trials were 5(93) and 6 (94) respectively, due to the lack of blind evaluation, follow-up and loss to follow up, and the inadequate reports of confounders adjustment. The details of the assessment are presented in Supplementary Tables 6, 7.

#### Effectiveness of Dietary Nutrients

Four studies all showed the cardioprotective effects of the dietary nutrient used in the trials. El Amrousy et al. (91) randomly divided the children with newly diagnosed ALL into two groups of 30 each. Children in intervention group received 1,000 mg omega 3 fatty acids capsule per day after the administration of DOX for 6 months, and children in control group received the DOX alone. The left ventricular systolic function was preserved in children who took omega 3 for 6 months, while the children in control group experienced significant impairments of cardiac function. Similarly, significantly lower MDA level and higher GSH and SOD levels of children in intervention group revealed that omega 3 fatty acids could decrease the early cardiac damage induced by DOX. Hagag et al. (92) recruited 40 ALL pediatric patients under DOX therapy, including 20 patients treated with black seed oil campus for 1 week and 20 patients treated with equivalent dose of placebo for the same amount of time. A larger reduction in parameters of systolic function arose in children with placebo compared to those with black seed oil. Iarussi et al. (93) carried a controlled trial on 20 children with ALL treated with anthracyclines, consisting of 10 patients with CoQ10 oral therapy and 10 without. Septum wall motion abnormalities were only detected in patients without CoQ10, which demonstrated prophylactic effects of CoQ10 on myocardial function from chemotherapeutic cardiotoxicity. Cortes et al. (94) enrolled 93 consecutive patients with advanced carcinoma to detect DOX-induced cardiotoxicity and the protective effects of CoQ10. Only 10 patients treated with DOX alone for more than 5 months and 8 patients treated with DOX and CoQ10 for more than 5 months were evaluated by systolic time intervals (STI). The mean of serial STIs in ten patients with DOX alone gradually increased during the course of DOX therapy and two patients had CHF. However, STIs were improved in eight patients with DOX and CoQ10 and only one patient had CHF. The effectiveness of dietary nutrients and the details of outcomes are summarized in Supplementary Figure 8 and Supplementary Table 8.

## Discussion

Our systematic review included 57 studies published in 14 countries from 1978 to 2021 consisting of 53 animal studies and four human studies, and summarized the cardioprotective effects of dietary nutrients derived from food on target subjects treated with chemotherapy. The descriptive synthetic evidence demonstrated that seven types of dietary nutrients (polyphenols, allicin, lycopene, PUFA, amino acids, CoQ10, and trace elements) might alleviate cardiovascular toxicity induced by chemotherapeutic agents.

As post-mitotic cells, cardiomyocytes are more sensitive to free radical damage due to their high oxidative metabolism and low antioxidant defense level (24). As a result, clinical and subclinical cardiac injuries related with chemotherapy have been a notorious issue. The incidence rates of CHF caused by anthracyclines and cyclophosphamides range 0.14–48% and 7–28%, respectively (6, 95). The childhood cancer survivors are 15 and 10 times more likely to suffer CHF and coronary artery disease, respectively than their siblings (96, 97). As early as in 1967, Tan et al. (98) first described the anthracycline-induced cardiotoxicity and reported that the development of tachycardia, arrhythmia and CHF in daunomycin patients could be associated with daunomycin. Simultaneously, it was found that cardiovascular toxicity was dose-dependent with a 5% incidence of cardiomyopathy at a cumulative dose of 400 mg/m^2^ of anthracyclines, 26% at a cumulative dose of 550 mg/m^2^ and up to 48% at 700 mg/m^2^ (99). That is the reason why the recommended cumulative dose is limited to 450–500 mg/m^2^ (100). Currently, cardiotoxic effects led by anthracyclines, especially DOX, have been most thoroughly studied (7, 19). And this is consistent with our review, in which 90.6% of the included animal studies generated cardiac dysfunctions by the injection of DOX in rats/mice. The most widely proposed mechanism is the anthracyclines' inhibition of topoisomerase 2β, which leads to promote cell apoptosis and generate oxidative damage in cardiomyocytes (13). At present, cardiotoxicity is a broader term without a formal definition (7, 101). The American Society of Echocardiography defines it as a ≥10% drop of LVEF from baseline or the absolute value <53% (101). 2016 European Society of Cardiology Position Paper considers the lower limit of normal LVEF as 50% (102). A clinical trial conducted on pediatric patients with acute myeloid leukemia also defined cardiotoxicity as LVEF <50% on the basis of the National Cancer Institute Common Terminology Criteria for Adverse Events (version 3) definitions (8). However, significantly abnormal cardiac parameters were considered as cardiotoxicity in most of the studies included in our review. Similarly, improved measurements or even back to normal was recognized as the signs of positive cardioprotective effects of nutritional intervention.

The relevant guideline recommended that oncologists considered prevention against chemotherapy-induced cardiotoxicity through long-term management during the early stage of anticancer treatment with support from cardiologists (6). Compared with dexrazoxane, dietary nutrition is more accessible at ordinary times and easier to comply with in long-term survivals. In other words, it can meet the two main advantages of daily and long-term usage. Consequently, it is an additional prevention measure that cancer survivors cannot miss. While the nutritional support has been depicted to improve the adverse effects of chemotherapy (103–105), the current evidence against cardiotoxicity was limited due to the lack of enough clinical studies (29, 30). In addition, the guidelines did not report in detail the aspect of nutrition against cardiomyopathy associated with cancer chemotherapy (6, 20, 96). Several published reviews introduced the application of nutritional intervention in the prevention of cardiac toxicity and covered CoQ10, grape seed extract and ω-3 PUFA (27, 28, 106). But none of them systematically summarized the effectiveness of overall dietary nutrients in this respect. Koss-Mikołajczyk et al. (29) showed that natural products (including fruit, vegetables, herbs, mushrooms, and phytochemicals) could counteract cardiac injury caused by DOX. Despite the comprehensive list of products included in this study, these were all edible plant extracts and foodborne phytochemicals. Nutrients derived from animal food as cardioprotective agents have not been explored. Therefore, our review summarized the current available evidence and filled in the corresponding gaps.

Seven types of dietary nutrients were represented in our review. Among them, polyphenols were in more than half of the included studies possibly due to more than 8,000 species in nature (including flavonoids and non-flavonoids) (107, 108). Polyphenols can eliminate oxygen free radicals by owning multiple PhOH (107) and the oxidation resistance has also made itself as a toxicity-related preventive strategy in some reviews (24, 26, 109). Thus, the extract of fresh fruit (rich in flavonols and flavonoids), grape seeds (rich in proanthocyanidin), and green tea (rich in catechins) were commonly used to ameliorate the chemotherapy-related cardiac damage in the included studies. Besides vegetable food, animal food was also made clear to protect the heart exposed to chemotherapy. PUFA are dietary factors with multiple beneficial effects and could likely protect cardiovascular tissues by adjusting cellular processes and molecular pathways (27, 110). The amino acids can preserve myocardial high-energy phosphate levels and prevent lactate accumulation. Our study refers to glutamine and glycine, which involve GSH synthesis (a vital intracellular antioxidant) (111). CoQ10 is a free-radical scavenger primarily present in metabolically active organs, such as the heart, liver, and kidney (82). Zn has a critical role in maintaining health, primarily through antioxidative stress and anti-inflammation, by catalyzing more than 300 enzymes and binding with over 2,500 proteins. Se prevents oxidative stress and maintains antioxidant enzymes such as the four glutathione peroxidases (GPx) (112, 113).

There were several limitations in our systematic review. First, 93.0% of the included records (53/57) were animal studies along with a relatively moderate to high risk of bias, so the interpretation of results should be more cautious. Second, our findings may still remain a certain distance approaching the clinical application due to the majority of the included studies being animal research. Third, due to the lack of standardization in definition of cardiotoxicity and the high heterogeneity from the variations of included studies, it was a pity that we couldn't provide pooled results in our review.

## Conclusion

Early prevention and management of cancer chemotherapy-induced cardiotoxicity have been increasingly focused due to the attention to event-free survival during and after cancer therapy. The existing studies have indicated that cardiotoxicity not only puts the patients under high risk of suffering cardiac deterioration but also develops as a social issue concerning the increase of Health System spending (114). The evidence of dietary nutrients against cardiovascular toxicity was still lacking. Our systematic review demonstrated that dietary nutrients (comprising polyphenols, allicin, lycopene, PUFA, amino acids, CoQ10, Zn, and Se) may be a potential strategy to protect cardiovascular system exposed to the chemotherapeutic agents, but more human studies are needed in future. On this basis, the development of cardioprotective strategies for special population, like children, the pregnant, and the elderly, is now essential for the reason that their vulnerable physical conditions demand much more cardiac protection.

## Data Availability Statement

The original contributions presented in the study are included in the article/Supplementary Material, further inquiries can be directed to the corresponding author/s.

## Author Contributions

K-WJ, QW, and X-NL contributed to the conception and design of the study. X-YZ and K-LY carried out the search strategy independently and wrote the draft manuscript. YL and YZ contributed to the analysis of the included studies. All authors are responsible for the final content of the manuscript and approved the final manuscript.

## Funding

This work was financially supported by Disciplinary Booster Program of Xijing Hospital, China (Project Nos. XJZT21CM27, XJZT19X11, and XJZT18Z22).

## Conflict of Interest

The authors declare that the research was conducted in the absence of any commercial or financial relationships that could be construed as a potential conflict of interest.

## Publisher's Note

All claims expressed in this article are solely those of the authors and do not necessarily represent those of their affiliated organizations, or those of the publisher, the editors and the reviewers. Any product that may be evaluated in this article, or claim that may be made by its manufacturer, is not guaranteed or endorsed by the publisher.
