# Supplementary material for: Can Dietary Nutrients Prevent Cancer Chemotherapy-Induced Cardiotoxicity? An Evidence Mapping of Human Studies and Animal Models
Source: Front Cardiovasc Med. 2022 Jun 29;9:921609. doi: 10.3389/fcvm.2022.921609 (PMC9277029; doi:10.3389/fcvm.2022.921609)
Supplement: Supplementary file 2 [file Data_Sheet_2.docx]

***Supplementary Material***

**Supplementary Figure 1 The effectiveness of polyphenols against cardiotoxicity in the animal studies**

CK, creatine kinase; CK-MB, creatine kinase-MB; DOX, doxorubicin; EGCG, Epigallocatechin-3-gallate; LDH, lactate dehydrogenase; LVEF, left ventricular ejection fraction; MDA, malondialdehyde; RESV, resveratrol; SOD, superoxide dismutase.

**Supplementary Figure 2 The effectiveness of allicin against cardiotoxicity in the animal studies**

CK-MB, creatine kinase-MB; DOX, doxorubicin; MDA, malondialdehyde; SOD, superoxide dismutase.

**Supplementary Figure 3 The effectiveness of lycopene against cardiotoxicity in the animal studies**

DOX, doxorubicin; MDA, malondialdehyde.

**Supplementary Figure 4 The effectiveness of polyunsaturated fatty acids against cardiotoxicity in the animal studies**

CK-MB, creatine kinase-MB; DOX, doxorubicin; GSH, malondialdehyde; LVEF, left ventricular ejection fraction; PUFA, polyunsaturated fatty acids; TRZ, trastuzumab.

**Supplementary Figure 5 The effectiveness of amino acids against cardiotoxicity in the animal studies**

DOX, doxorubicin; FOLFOW, 5-fluorouracil+leucovorin+oxaliplatin; GSH, malondialdehyde; LVEF, left ventricular ejection fraction.

**Supplementary Figure 6 The effectiveness of coenzyme Q10 against cardiotoxicity in the animal studies**

CK, creatine kinase; CoQ10, coenzyme Q10; DOX, doxorubicin; MDA, malondialdehyde.

**Supplementary Figure 7 The effectiveness of trace elements against cardiotoxicity in the animal studies**

CK-MB, creatine kinase-MB; cTn, cardiac troponin; DOX, doxorubicin; MDA, malondialdehyde; ZnCM, zinc+curcumin.

**Supplementary Figure 8 The effectiveness of dietary nutrients against cardiotoxicity in the human studies**

CoQ10, coenzyme Q10; cTn, cardiac troponin; DOX, doxorubicin; LVEF, left ventricular ejection fraction; LVFS, left ventricular fractional shortening; PUFA, polyunsaturated fatty acids; STI, systolic time interval.

**Supplementary Table 1 Category, abbreviation and function of detection indicators**

| **Category** | **Indicators** | **Abbreviation** | **Clinical Significance** | **Change indicating the cardiotoxicity** |
| --- | --- | --- | --- | --- |
| Echocardiography | left ventricular ejection fraction | LVEF | stroke volume/left ventricular end-diastolic volume, reflecting myocardial contractility | **↓** |
|  | left ventricular fractional shortening | LVFS | (LVEED-LVESD)/LVEED, reflecting myocardial contractility | **↓** |
|  | left ventricular end-systolic diameter | LVESD | left ventricular dilation, reflecting reduced left ventricular function | **↑** |
|  | left ventricular end-diastolic diameter | LVEED | left ventricular dilation, reflecting reduced left ventricular function | **↑** |
| Serum cardiac markers | creatine kinase | CK | mainly in skeletal muscle, brain and myocardium, and its rise indicating acute myocardial infarction and myocardial ischemia | **↑** |
|  | creatine kinase-MB | CK-MB | mainly in myocardial cell, and its rise indicating myocardial infarction | **↑** |
|  | lactate dehydrogenase | LDH | a vital oxidordeuctase mainly in myocardium, liver and kidney, and its rise indicating myocardial infarction | **↑** |
|  | aspartate transaminase | AST | mainly in mitochondria of cardiomyocytes and hepatocytes, and lots of release in the blood when cardiomyocytes injury | **↑** |
|  | brain natriuretic peptide | BNP | a neurohormone secreted by the ventricle, and its rise indicating left ventricular dysfunction | **↑** |
|  | N-terminal pro-brain natriuretic peptide | NT-proBNP | one of the precursors of brain natriuretic peptide | **↑** |
|  | cardiac troponin | cTn | a protein regulating contraction of myocardium, and its release in the blood when cardiomyocytes injury | **↑** |
| Oxidative stress markers | malondialdehyde | MDA | the end product of lipid oxidation and reflecting the degree of lipid peroxidation | **↑** |
|  | superoxide dismutase | SOD | an important antioxidant enzyme and keeping balance between oxidation and anti-oxidation | **↓** |
|  | glutathione | GSH | maintaining immune and antioxidant effects | **↓** |
|  | glutathione peroxidase | GPx | an important peroxide-decomposing enzyme to avoid oxidative damage | **↓** |
|  | catalase | CAT | an enzyme that catalyzes the breakdown of hydrogen peroxide into oxygen and water and avoids oxidative damage | **↓** |

**Supplementary Table 2 Search strategies**

**2.1 Medline (via PubMed) Search Strategy**

Search Date: 9 Nov 2021

| **Search** | **Query** |
| --- | --- |
| **#1** | "Drug Therapy"[MeSH] |
| **#2** | drug therapy[Title/Abstract] OR chemotherap*[Title/Abstract] OR antineoplastic protocol*[Title/Abstract] OR cancer treatment protocol*[Title/Abstract] OR antineoplastic agent*[Title/Abstract] OR antineoplastic drug*[Title/Abstract] OR anticancer drug*[Title/Abstract] OR Pharmacotherap*[Title/Abstract] OR Anthracycline*[Title/Abstract] OR Doxorubicin[Title/Abstract] OR Adriamycin[Title/Abstract] |
| **#3** | #1 OR #2 |
| **#4** | "Neoplasms"[MeSH] |
| **#5** | Neoplasm*[Title/Abstract] OR Neoplasia*[Title/Abstract] OR Neoplastic[Title/Abstract] OR Tumor*[Title/Abstract] OR Tumour*[Title/Abstract] OR Cancer*[Title/Abstract] OR Malignanc*[Title/Abstract] |
| **#6** | #4 OR #5 |
| **#7** | "Heart Diseases"[MeSH] |
| **#8** | Heart Disease*[Title/Abstract] OR Heart Disorder*[Title/Abstract] OR Heart deficiency[Title/Abstract] OR Heart deformity[Title/Abstract] OR Cardiac Disease*[Title/Abstract] OR Cardiac Disorder*[Title/Abstract] OR Cardiac Anomaly[Title/Abstract] OR Cardiac Disturbance[Title/Abstract] OR Cardiopathy[Title/Abstract] OR Cardiotoxicit*[Title/Abstract] OR Cardiac Toxicit*[Title/Abstract] OR Cardio Toxicit*[Title/Abstract] OR Cardiotoxic effect[Title/Abstract] OR Cardiotoxicology[Title/Abstract] OR Heart toxicity[Title/Abstract] OR Heart Failure[Title/Abstract] OR Cardiomyopathy[Title/Abstract] OR Cardiac Function[Title/Abstract] OR Ventricular dysfunction[Title/Abstract] OR Heart dysfunction[Title/Abstract] |
| **#9** | #7 OR #8 |
| **#10** | "Nutrition Therapy"[MeSH] OR "Diet"[MeSH] |
| **#11** | Nutrition*[Title/Abstract] OR Nutrition* Therapy[Title/Abstract] OR Nutrition* Support[Title/Abstract] OR Nutrition* Intervention*[Title/Abstract] OR Enteral Nutrition[Title/Abstract] OR Parenteral Nutrition[Title/Abstract] OR Diet*[Title/Abstract] OR Dietary[Title/Abstract] OR Oral Nutritional Supplement*[Title/Abstract] OR Oral Supplement*[Title/Abstract] OR Oral Nutrition*[Title/Abstract] OR Food[Title/Abstract] OR diet, food, and nutrition[Title/Abstract] OR Energy intake[Title/Abstract] OR Glutamine[Title/Abstract] OR Omega-3 Polyunsaturated Fatty Acids[Title/Abstract] OR Fatty Acids, Omega-3[Title/Abstract] OR Nutrient*[Title/Abstract] OR Macronutrient*[Title/Abstract] OR Micronutrient*[Title/Abstract] |
| **#12** | #10 OR #11 |
| **#13** | #3 AND #6 AND #9 AND #12 |

**2.2 Embase Search Strategy**

Search Date: 9 Nov 2021

| **Search** | **Query** |
| --- | --- |
| **#1** | 'chemotherapy'/exp |
| **#2** | 'drug therapy':ti,ab OR 'chemotherap*':ti,ab OR 'antineoplastic protocol*':ti,ab OR 'cancer treatment protocol*':ti,ab OR 'antineoplastic agent*':ti,ab OR 'antineoplastic drug*':ti,ab OR 'anticancer drug*':ti,ab OR 'pharmacotherap*':ti,ab OR 'anthracycline*':ti,ab OR 'doxorubicin':ti,ab OR 'adriamycin':ti,ab |
| **#3** | #1 OR #2 |
| **#4** | 'neoplasm'/exp |
| **#5** | neoplasm*:ti,ab OR neoplasia*:ti,ab OR neoplastic:ti,ab OR tumor*:ti,ab OR tumour*:ti,ab OR cancer*:ti,ab OR malignanc*:ti,ab |
| **#6** | #4 OR #5 |
| **#7** | 'heart disease'/exp |
| **#8** | 'heart disease*':ti,ab OR 'heart disorder*':ti,ab OR 'heart deficiency':ti,ab OR 'heart deformity':ti,ab OR 'cardiac disease*':ti,ab OR 'cardiac disorder*':ti,ab OR 'cardiac anomaly':ti,ab OR 'cardiac disturbance':ti,ab OR cardiopathy:ti,ab OR cardiotoxicit*:ti,ab OR 'cardiac toxicit*':ti,ab OR 'cardio toxicit*':ti,ab OR 'cardiotoxic effect':ti,ab OR cardiotoxicology:ti,ab OR 'heart toxicity':ti,ab OR 'heart failure':ti,ab OR cardiomyopathy:ti,ab OR 'cardiac function':ti,ab OR 'ventricular dysfunction':ti,ab OR 'heart dysfunction':ti,ab |
| **#9** | #7 OR #8 |
| **#10** | 'nutrition'/exp OR 'diet therapy'/exp |
| **#11** | nutrition*:ti,ab OR 'nutrition* therapy':ti,ab OR 'nutrition* support':ti,ab OR 'nutrition* intervention*':ti,ab OR 'enteral nutrition':ti,ab OR 'parenteral nutrition':ti,ab OR diet*:ti,ab OR dietary:ti,ab OR 'oral nutritional supplement*':ti,ab OR 'oral supplement*':ti,ab OR 'oral nutrition*':ti,ab OR food:ti,ab OR 'diet, food, and nutrition':ti,ab OR 'energy intake':ti,ab OR glutamine:ti,ab OR 'omega-3 polyunsaturated fatty acids':ti,ab OR 'fatty acids, omega-3':ti,ab OR nutrient*:ti,ab OR macronutrient*:ti,ab OR micronutrient*:ti,ab |
| **#12** | #10 OR #11 |
| **#13** | #3 AND #6 AND #9 AND #12 |

**2.3 Cochrane Library Search Strategy**

Search Date:9 Nov 2021

| **Search** | **Query** |
| --- | --- |
| **#1** | MeSH descriptor: [Drug Therapy] explode all trees |
| **#2** | (drug therapy):ti,ab,kw OR (chemotherap*):ti,ab,kw OR (antineoplastic protocol*):ti,ab,kw OR (cancer treatment protocol*):ti,ab,kw OR (antineoplastic agent*):ti,ab,kw OR (antineoplastic drug*):ti,ab,kw OR (anticancer drug*):ti,ab,kw OR (Pharmacotherap*):ti,ab,kw OR (Anthracycline*):ti,ab,kw OR (Doxorubicin):ti,ab,kw OR (Adriamycin):ti,ab,kw |
| **#3** | #1 OR #2 |
| **#4** | MeSH descriptor: [Neoplasms] explode all trees |
| **#5** | (Neoplasm*):ti,ab,kw OR (Neoplasia*):ti,ab,kw OR (Neoplastic):ti,ab,kw OR (Tumor*):ti,ab,kw OR (Tumour*):ti,ab,kw OR (Cancer*):ti,ab,kw OR (Malignanc*):ti,ab,kw |
| **#6** | #4 OR #5 |
| **#7** | MeSH descriptor: [Heart Diseases] explode all trees |
| **#8** | (Heart Disease*):ti,ab,kw OR (Heart Disorder*):ti,ab,kw OR (Heart deficiency):ti,ab,kw OR (Heart deformity):ti,ab,kw OR (Cardiac Disease*):ti,ab,kw OR (Cardiac Disorder*):ti,ab,kw OR (Cardiac Anomaly):ti,ab,kw OR (Cardiac Disturbance):ti,ab,kw OR (Cardiopathy):ti,ab,kw OR (Cardiotoxicit*):ti,ab,kw OR (Cardiac Toxicit*):ti,ab,kw OR (Cardio Toxicit*):ti,ab,kw OR (Cardiotoxic effect):ti,ab,kw OR (Cardiotoxicology):ti,ab,kw OR (Heart toxicity):ti,ab,kw OR (Heart Failure):ti,ab,kw OR (Cardiomyopathy):ti,ab,kw OR (Cardiac Function):ti,ab,kw OR (Ventricular dysfunction):ti,ab,kw OR (Heart dysfunction):ti,ab,kw |
| **#9** | #7 OR #8 |
| **#10** | "Nutrition Therapy"[MeSH] OR "Diet"[MeSH] |
| **#11** | (Nutrition*):ti,ab,kw OR (Nutrition* Therapy):ti,ab,kw OR (Nutrition* Support):ti,ab,kw OR (Nutrition* Intervention*):ti,ab,kw OR (Enteral Nutrition):ti,ab,kw OR (Parenteral Nutrition):ti,ab,kw OR (Diet*):ti,ab,kw OR (Dietary):ti,ab,kw OR (Oral Nutritional Supplement*):ti,ab,kw OR (Oral Supplement*):ti,ab,kw OR (Oral Nutrition*):ti,ab,kw OR (Food):ti,ab,kw OR (diet, food, and nutrition):ti,ab,kw OR (Energy intake):ti,ab,kw OR (Glutamine):ti,ab,kw OR (Omega-3 Polyunsaturated Fatty Acids):ti,ab,kw OR (Fatty Acids, Omega-3):ti,ab,kw OR (Nutrient*):ti,ab,kw OR (Macronutrient*):ti,ab,kw OR (Micronutrient*):ti,ab,kw |
| **#12** | #10 OR #11 |
| **#13** | #3 AND #6 AND #9 AND #12 |

**Supplementary Table 3 Reasons for key excluded reviews**

| **Studies** | **Excluded reasons** |
| --- | --- |
| (1,2) | Irrelevant participants (n=2) |
| (3–26) | Inappropriate intervention (n=24) |
| (27,28) | Wrong design (n=2) |
| (29–37) | Irrelevant outcomes (n=9) |

1. Cheung KG, Cole LK, Xiang B, Chen K, Ma X, Myal Y, Hatch GM, Tong Q, Dolinsky VW. Sirtuin-3 (SIRT3) Protein Attenuates Doxorubicin-induced Oxidative Stress and Improves Mitochondrial Respiration in H9c2 Cardiomyocytes. *J Biol Chem* (2015) **290**:10981–10993. doi: 10.1074/jbc.M114.607960

2. Aleksandar P, Dragana M-Ć, Nebojša J, Biljana N, Nataša S, Branka V, Jelena K-V. Wild edible onions — Allium flavum and Allium carinatum — successfully prevent adverse effects of chemotherapeutic drug doxorubicin. *Biomed Pharmacother* (2019) **109**:2482–2491. doi: 10.1016/j.biopha.2018.11.106

3. Albini A, Festa MMG, Ring N, Baci D, Rehman M, Finzi G, Sessa F, Zacchigna S, Bruno A, Noonan DM. A Polyphenol-Rich Extract of Olive Mill Wastewater Enhances Cancer Chemotherapy Effects, While Mitigating Cardiac Toxicity. *Front Pharmacol* (2021) **12**:694762. doi: 10.3389/fphar.2021.694762

4. Irrera N, Pallio G, Mannino F, Gugliotta R, Metro D, Altavilla D, Squadrito F. Administration of a Nutraceutical Mixture Composed by *Aloe arborescens* , *Annona muricata* , *Morinda citrifolia* , *Beta rubra* , *Scutellaria baicalensis* , and *Vaccinium myrtillus* Reduces Doxorubicin-Induced Side Effects. *Nutr Cancer* (2020) **72**:343–351. doi: 10.1080/01635581.2019.1633364

5. Du X, Schelegle E, Mohr FC, Margolin SB, Giri SN, Al-Bayati MA. Amelioration of doxorubicin-induced cardiac and renal toxicity by pirfenidone in rats. *Cancer Chemother Pharmacol* (2004) **53**:141–150. doi: 10.1007/s00280-003-0703-z

6. Jagetia GC, Reddy TK, Malagi KJ, Nayak BS, Naidu MBR, Ravikiran PB, Kamath SU, Shetty PC, Reddy DS. Antarth, a polyherbal preparation protects against the doxorubicin-induced toxicity without compromising its Antineoplastic activity. *Phytother Res* (2005) **19**:772–778. doi: 10.1002/ptr.1713

7. Carresi C, Musolino V, Gliozzi M, Maiuolo J, Mollace R, Nucera S, Maretta A, Sergi D, Muscoli S, Gratteri S, et al. Anti-oxidant effect of bergamot polyphenolic fraction counteracts doxorubicin-induced cardiomyopathy: Role of autophagy and c-kitposCD45negCD31neg cardiac stem cell activation. *J Mol Cell Cardiol* (2018) **119**:10–18. doi: 10.1016/j.yjmcc.2018.04.007

8. Dolinsky VW, Rogan KJ, Sung MM, Zordoky BN, Haykowsky MJ, Young ME, Jones LW, Dyck JRB. Both aerobic exercise and resveratrol supplementation attenuate doxorubicin-induced cardiac injury in mice. *Am J Physiol-Endocrinol Metab* (2013) **305**:E243–E253. doi: 10.1152/ajpendo.00044.2013

9. Chakraborty M, Bhattacharjee A, Kamath JV. Cardioprotective effect of ursolic acid against doxorubicin induced cardiotoxicity. *Indian Drugs* (2016) **53**:65–71.

10. Olorundare OE, Adeneye AA, Akinsola AO, Sanni DA, Koketsu M, Mukhtar H. Clerodendrum volubile</i> Ethanol Leaf Extract: A Potential Antidote to Doxorubicin-Induced Cardiotoxicity in Rats. *J Toxicol* (2020) **2020**:1–17. doi: 10.1155/2020/8859716

11. Cote B, Carlson LJ, Rao DA, Alani AWG. Combinatorial resveratrol and quercetin polymeric micelles mitigate doxorubicin induced cardiotoxicity in vitro and in vivo. *J Controlled Release* (2015) **213**:128–133. doi: 10.1016/j.jconrel.2015.06.040

12. Zhu S-G, Kukreja RC, Das A, Chen Q, Lesnefsky EJ, Xi L. Dietary Nitrate Supplementation Protects Against Doxorubicin-Induced Cardiomyopathy by Improving Mitochondrial Function. *J Am Coll Cardiol* (2011) **57**:2181–2189. doi: 10.1016/j.jacc.2011.01.024

13. Mong M, Hsia T, Yin M. Dietary Trans Fats Enhance Doxorubicin-Induced Cardiotoxicity in Mice: Cardiotoxicity of trans fat…. *J Food Sci* (2013) **78**:H1621–H1628. doi: 10.1111/1750-3841.12257

14. El-Demerdash E, Ali AA, El-Taher DEM, Hamada FMA. Effect of low-protein diet on anthracycline pharmacokinetics and cardiotoxicity. *J Pharm Pharmacol* (2012) **64**:344–352. doi: 10.1111/j.2042-7158.2011.01413.x

15. Breitbart E, Lomnitski L, Nyska A, Malik Z, Bergman M, Sofer Y, Haseman JK, Grossman S. Effects of water-soluble antioxidant from spinach, NAO, on doxorubicin-induced heart injury. *Hum Exp Toxicol* (2001) **20**:337–345. doi: 10.1191/096032701680350604

16. Toblli JE, Rivas C, Cao G, Giani JF, Funk F, Mizzen L, Dominici FP. Ferric Carboxymaltose-Mediated Attenuation of Doxorubicin-Induced Cardiotoxicity in an Iron Deficiency Rat Model. *Chemother Res Pract* (2014) **2014**:1–9. doi: 10.1155/2014/570241

17. Abo Mansour HE, El-Batsh MM, Badawy NS, Mehanna ET, Mesbah NM, Abo-Elmatty DM. Ginger Extract Loaded into Chitosan Nanoparticles Enhances Cytotoxicity and Reduces Cardiotoxicity of Doxorubicin in Hepatocellular Carcinoma in Mice. *Nutr Cancer* (2021) **73**:2347–2362. doi: 10.1080/01635581.2020.1823436

18. Li L, Ni J, Li M, Chen J, Han L, Zhu Y, Kong D, Mao J, Wang Y, Zhang B, et al. Ginsenoside Rg3 micelles mitigate doxorubicin-induced cardiotoxicity and enhance its anticancer efficacy. *Drug Deliv* (2017) **24**:1617–1630. doi: 10.1080/10717544.2017.1391893

19. Xue H, Ren W, Denkinger M, Schlotzer E, Wischmeyer PE. Nutrition Modulation of Cardiotoxicity and Anticancer Efficacy Related to Doxorubicin Chemotherapy by Glutamine and ω-3 Polyunsaturated Fatty Acids. *J Parenter Enter Nutr* (2016) **40**:52–66. doi: 10.1177/0148607115581838

20. Abdelghffar EA, El-Nashar HAS, AL-Mohammadi AGA, Eldahshan OA. Orange fruit ( *Citrus sinensis* ) peel extract attenuates chemotherapy-induced toxicity in male rats. *Food Funct* (2021) **12**:9443–9455. doi: 10.1039/D1FO01905H

21. Nagai K, Konishi H. Protection of theanine against doxorubicin-induced acute cardiac toxicity. *Biomed Prev Nutr* (2013) **3**:197–199. doi: 10.1016/j.bionut.2013.03.011

22. Sadzuka Y, Sugiyama T, Shimoi K, Kinae N, Hirota S. Protective effect of flavonoids on doxorubicin-induced cardiotoxicity. *Toxicol Lett* (1997) **92**:1–7. doi: 10.1016/S0378-4274(97)00028-3

23. Link G, Tirosh R, Pinson A, Hershko C. Role of iron in the potentiation of anthracycline cardiotoxicity: Identification of heart cell mitochondria as a major site of iron-anthracycline interaction. *J Lab Clin Med* (1996) **127**:272–278. doi: 10.1016/S0022-2143(96)90095-5

24. Mostafa MG, Mima T, Ohnishi ST, Mori K. S-Allylcysteine Ameliorates Doxorubicin Toxicity in the Heart and Liver in Mice. *Planta Med* (2000) **66**:148–151. doi: 10.1055/s-2000-11124

25. Kihara M, Kaiya H, Hirai Y, Katayama H, Terao A, Nishikawa M. Salmon acyl-ghrelin increases food intake and reduces doxorubicin-induced myocardial apoptosis in rats, likely by anti-oxidative activity. *Peptides* (2021) **137**:170471. doi: 10.1016/j.peptides.2020.170471

26. Karabulut D, Ozturk E, Kaymak E, Akin AT, Yakan B. Thymoquinone attenuates doxorubicin‐cardiotoxicity in rats. *J Biochem Mol Toxicol* (2021) **35**: doi: 10.1002/jbt.22618

27. Harvey PA, Leinwand LA. Dietary phytoestrogens present in soy dramatically increase cardiotoxicity in male mice receiving a chemotherapeutic tyrosine kinase inhibitor. *Mol Cell Endocrinol* (2015) **399**:330–335. doi: 10.1016/j.mce.2014.10.011

28. Alfaro Y, Delgado G, Cárabez A, Anguiano B, Aceves C. Iodine and doxorubicin, a good combination for mammary cancer treatment: antineoplastic adjuvancy, chemoresistance inhibition, and cardioprotection. *Mol Cancer* (2013) **12**:45. doi: 10.1186/1476-4598-12-45

29. Bishop S, Liu SJ. Cardioprotective action of the aqueous extract of Terminalia arjuna bark against toxicity induced by doxorubicin. *Phytomedicine* (2017) **36**:210–216. doi: 10.1016/j.phymed.2017.10.007

30. Raghad K, Ljubory I, Rahim S. Cardioprotective effect of alcohol extract and seed powder dietary supplementation of quinoa (Chenopodium quinoa) in female rats treated with Doxorubicin. *J Cardiovasc Dis Res* (2021) **12**:157–163.

31. Wergeland A, Bester DJ, Sishi BJN, Engelbrecht AM, Jonassen AK, Van Rooyen J. Dietary red palm oil protects the heart against the cytotoxic effects of anthracycline: ANTIOXIDANT-RICH OIL AGAINST CARDIOTOXICITY. *Cell Biochem Funct* (2011) **29**:356–364. doi: 10.1002/cbf.1756

32. Cusack BJ, Young SP, Loseke VL, Hurty MR, Beals L, Olson RD. Effect of a low-protein diet on doxorubicin pharmacokinetics in the rabbit. *Cancer Chemother Pharmacol* (1992) **30**:145–148. doi: 10.1007/BF00686407

33. Parabathina R, Muralinath E, Lakshmana Swamy P, Hari Krishna V, Shanthi Sree K. Effects of vitamin-E, morin, rutin, quercetin against doxorubicin in rabbits: A hematological study. *Res J Pharm Biol Chem Sci* (2011) **2**:74–84.

34. Sudharsan PT, Mythili Y, Selvakumar E, Varalakshmi P. Lupeol and its ester ameliorate the cyclophosphamide provoked cardiac lysosomal damage studied in rat. *Mol Cell Biochem* (2006) **282**:23–29. doi: 10.1007/s11010-006-1169-1

35. Sudharsan PT, Mythili Y, Selvakumar E, Varalakshmi P. Lupeol and its ester inhibit alteration of myocardial permeability in cyclophosphamide administered rats. *Mol Cell Biochem* (2006) **292**:39–44. doi: 10.1007/s11010-006-9171-1

36. Stewart LK, Smoak P, Hydock DS, Hayward R, O’Brien K, Lisano JK, Boeneke C, Christensen M, Mathias A. Milk and kefir maintain aspects of health during doxorubicin treatment in rats. *J Dairy Sci* (2019) **102**:1910–1917. doi: 10.3168/jds.2018-15576

37. Coudray C, Hida H, Boucher F, De Leiris J, Favier A. Modulation by selenium supplementation of lipid peroxidation induced by chronic administration of adriamycin in rats. *Nutr Burbank Los Angel Cty Calif* (1995) **11**:512–516.

**Supplementary Table 4 Risk of bias assessment of included animal studies using the risk of bias tool of Systematic Review Center for Laboratory Animal Experimentation**

| **Studies** | **Sequence generation** | **Baseline characteristics** | **Allocation concealment** | **Random housing** | **Performance blinding** | **Random outcome assessment** | **Blinding of outcome assessment** | **Incomplete outcome data** | **Selective outcome reporting** | **Other sources of bias** |
| --- | --- | --- | --- | --- | --- | --- | --- | --- | --- | --- |
| **Adıyaman et al., 2021** | U | U | U | Y | U | U | U | Y | Y | Y |
| **Ammar et al., 2013** | U | U | U | Y | U | U | U | Y | Y | Y |
| **Boghdady, 2013** | U | U | U | Y | U | U | U | Y | Y | Y |
| **Yalcin et al., 2010** | U | U | U | Y | U | U | U | Y | Y | Y |
| **Yousef et al., 2009** | U | U | U | Y | U | U | U | Y | Y | Y |
| **Zhang et al., 2005** | U | U | U | U | U | U | U | U | Y | Y |
| **Petroni et al., 2017** | U | U | U | Y | U | U | U | N | Y | Y |
| **Shoukry et al., 2017** | U | U | U | Y | U | U | U | U | Y | Y |
| **Arafa et al., 2014** | U | U | U | Y | U | U | U | Y | Y | Y |
| **IbrahimFouad and Ahmed., 2021** | U | U | U | U | U | U | U | U | Y | Y |
| **Bahadır et al., 2018** | U | U | U | Y | U | U | U | Y | Y | Y |
| **Benzer et al., 2017** | U | U | U | Y | U | U | U | U | Y | Y |
| **Swamy et al., 2012** | U | U | U | Y | U | U | U | Y | Y | Y |
| **Venkatesan, 1998** | U | U | U | U | U | U | U | U | Y | Y |
| **Ibrahim et al., 2019** | U | U | U | Y | U | U | U | U | Y | Y |
| **Saeed et al., 2015** | U | U | U | Y | U | U | U | U | Y | Y |
| **Amanullah et al., 2015** | U | U | U | Y | U | U | U | U | Y | Y |
| **Mubarak et al., 2018** | U | U | U | Y | U | U | U | U | Y | Y |
| **Sabbah et al., 2018** | U | U | U | Y | U | U | U | U | Y | Y |
| **Ribeiro et al., 2021** | U | U | U | Y | U | U | U | U | Y | Y |
| **Elberry et al., 2010** | U | U | U | Y | U | U | U | N | Y | Y |
| **Abu Gazia and El-Magd, 2018** | U | U | U | Y | U | U | U | U | Y | Y |
| **Hassanpour Fard et al., 2011** | U | U | U | Y | U | U | U | U | Y | Y |
| **Shatoor and Said Ahmed, 2014** | U | U | U | Y | U | U | U | U | Y | Y |
| **Subburaman et al., 2014** | U | U | U | Y | U | U | U | Y | Y | Y |
| **Abdel-Wahab et al., 2003** | U | U | U | Y | U | U | U | U | Y | Y |
| **Alhumaydhi, 2020** | U | U | U | Y | U | U | U | U | Y | Y |
| **Abu-Elsaad et al., 2015** | U | U | U | Y | U | U | U | U | Y | Y |
| **Lin et al., 2019** | U | U | U | Y | U | U | U | U | Y | Y |
| **Abdel-Daim et al., 2017** | U | U | U | Y | U | U | U | Y | Y | Y |
| **Demirkaya et al., 2009** | U | U | U | Y | U | U | U | U | Y | Y |
| **Mukherjee et al., 2003** | U | U | U | Y | U | U | U | U | Y | Y |
| **Ferreira et al., 2007** | U | U | U | Y | U | U | U | N | Y | Y |
| **Yilmaz et al., 2006** | U | U | U | Y | U | U | U | U | Y | Y |
| **Ahmed et al., 2021** | U | U | U | Y | U | U | U | U | Y | Y |
| **Asselin et al., 2020** | U | Y | U | Y | U | U | U | U | Y | Y |
| **Saleh et al., 2020** | U | U | U | Y | U | U | U | U | Y | Y |
| **Saleem et al., 2014** | U | U | U | Y | U | U | U | Y | Y | Y |
| **Teng et al., 2010** | U | U | U | Y | U | U | U | N | Y | Y |
| **Maneikyte et al., 2020** | U | Y | U | Y | U | U | Y | N | Y | N |
| **Todorova et al., 2010a** | U | Y | U | Y | U | U | U | N | Y | Y |
| **Todorova et al., 2010b** | U | Y | U | Y | U | U | U | N | Y | Y |
| **Cao et al., 1999** | U | U | U | Y | U | U | U | U | Y | Y |
| **Rahmanifard et al., 2021** | U | U | U | Y | U | U | U | Y | Y | Y |
| **Shabaan et al., 2021** | U | U | U | Y | U | U | U | Y | Y | Y |
| **Botelho et al., 2020** | U | U | U | Y | U | U | U | Y | Y | Y |
| **Chen et al., 2017** | U | U | U | U | Y | U | U | Y | Y | Y |
| **Mustafa et al., 2017** | U | U | U | Y | U | U | U | U | Y | Y |
| **Maryoosh et al., 2020** | U | U | U | Y | U | U | U | Y | Y | Y |
| **Wu et al., 2019** | U | U | U | Y | U | U | U | Y | Y | Y |
| **Coudray et al., 1996** | U | U | U | Y | U | U | U | N | Y | Y |
| **Radeva-Ilieva et al., 2020** | U | U | U | Y | U | U | U | U | Y | Y |
| **Wahab et al., 2000** | U | U | U | U | U | U | U | U | Y | Y |

Y, low risk of bias; N, high risk of bias; U, unclear risk of bias.

**Supplementary Table 6 Risk of bias assessment of the included randomized controlled trials using the Cochrane risk of bias tool**

| **Studies** | **Sequence generation** | **Allocation concealment** | **Blinding of participants and personnel** | **Blinding of outcome assessment** | **Incomplete outcome data** | **Selective outcome reporting** | **Other biases** |
| --- | --- | --- | --- | --- | --- | --- | --- |
| **El Amrousy et al., 2021** | Y | Y | Y | Y | Y | Y | Y |
| **Hagag et al., 2020** | Y | Y | U | U | Y | U | Y |

Y, low risk of bias; N, high risk of bias; U, unclear risk of bias.

**Supplementary Table 7 Risk of bias assessment of the included non-randomized controlled trial using the Newcastle-Ottawa Scale**

| **Dimensions** | **Items** | **Iarussi et al., 1994** | **Cortes et al., 1978** |
| --- | --- | --- | --- |
| **Selection** | 1. **Representativeness of the Exposed Cohort** | 1 | 1 |
|  | 1. **Selection of the Non-Exposed Cohort** | 1 | 1 |
|  | 1. **Ascertainment of Exposure** | 1 | 1 |
|  | 1. **Demonstration That Outcome of Interest Was Not Present at Start of Study** | 1 | 1 |
| **Comparability** | 1. **Comparability of Cohorts on the Basis of the Design or Analysis** | 1 | 1 |
| **Outcome** | 1. **Assessment of Outcome** | 0 | 0 |
|  | 1. **Was Follow-Up Long Enough for Outcomes to Occur** | 0 | 1 |
|  | 1. **Adequacy of Follow Up of Cohorts** | 0 | 0 |

**Supplementary Table 8 Outcomes of included human studies**

| **Studies** | **Grouping** | | **Echocardiography** | | | | **Serum cardiac markers** | | | **Oxidative stress markers** | | | **Electrocardiograph** |
| --- | --- | --- | --- | --- | --- | --- | --- | --- | --- | --- | --- | --- | --- |
|  |  |  | **LVEF**  **(%)** | **LVFS**  **(%)** | **SWT**  **(%)** | **2D-GLS** | **CK-MB**  **(U/L)** | **cTn I**  **(ng/ml)** | **NT-proBNP**  **(pg/ml)** | **MDA**  **(ng/ml)** | **SOD**  **(U/L)** | **GSH**  **(mg/dl)** | **Systolic time interval**  **(%)** |
| El Amrousy et al., 2021 | ①DOX | Before |  | 40.7±5.2 |  | −19.8±1.2 | 11.6±3.6 | <001 | 37.1±7.7 | 7.5±1.3 | 4.3±0.8 | 25.4±3.3 |  |
|  |  | After |  | 36±3.8 |  | −15.8±1.6^*^ | 46 ±7.2^*^ | 0.050±0.012^*^ | 88.8±13.6^*^ | 9.7±1.2^*^ | 2.9±0.7^*^ | 20.6±1.4^*^ |  |
|  | ②DOX+omega 3 fatty acids | Before |  | 40±5.4 |  | −19.3±1.7 | 10.7±3.7 | <0.001 | 36.8±8.3 | 8±1.2 | 3.9±1 | 25±2.4 |  |
|  |  | After |  | 38.2±6.3 |  | −18.7±1.2^#^ | 12±2.1^#^ | 0.003±0.001^#^ | 393±9.8^#^ | 6.1±1.1^*#^ | 5.8±0.9^*#^ | 28.7±2.5^*#^ |  |
| Hagag et al., 2020 | ①DOX+placebo | Before | 48.25±3.91 | 36.1±3.6 |  |  |  |  |  |  |  |  |  |
|  |  | After | 47.5±5.99^*^ | 34.89±6.34^*^ |  |  |  |  |  |  |  |  |  |
|  | ②DOX+black seed oil | Before | 47.5±2.8 | 35.6±1.93 |  |  |  |  |  |  |  |  |  |
|  |  | After | 52.25±5.35^*#^ | 39.97±6.83^*#^ |  |  |  |  |  |  |  |  |  |
| Larussi et al., 1994 | ①anthracyclines | Before |  | 39.89±4.37 | 46,10±10.10 |  |  |  |  |  |  |  |  |
|  |  | After |  | 33.43±3.46^*^ | 27.00±18.54^*^ |  |  |  |  |  |  |  |  |
|  | ②anthracyclines+CoQ10 | Before |  | 40.36±4.60 | 44.10±13.20 |  |  |  |  |  |  |  |  |
|  |  | After |  | 35.82±5.02^*^ | 40.10±15.30 |  |  |  |  |  |  |  |  |
| Cortes et al., 1978 | ①DOX | Before |  |  |  |  |  |  |  |  |  |  | 0.348±0.02 |
|  |  | After |  |  |  |  |  |  |  |  |  |  | 0.367±0.02 |
|  | ②DOX+CoQ10 | Before |  |  |  |  |  |  |  |  |  |  | 0.360±0.01 |
|  |  | After |  |  |  |  |  |  |  |  |  |  | 0.302±0.01 |

2D-GLS, two-dimensional global longitudinal strain; CK-MB, creatine kinase MB; cTnI, cardiac troponin I; GSH, glutathione; LVEF, left ventricular ejection fraction; LVFS, left ventricular fractional shortening; MDA, malondialdehyde; NT-proBNP, N-terminal pro-brain natriuretic peptide; SOD, superoxide dismutase; SWT, septal wall thickening.

^*^statistically different from the baseline (p＜0.05); ^#^statistically different from DOX group (p＜0.05).
