# Supplementary figures and images for: Can Dietary Nutrients Prevent Cancer Chemotherapy-Induced Cardiotoxicity? An Evidence Mapping of Human Studies and Animal Models
Source: Front Cardiovasc Med. 2022 Jun 29;9:921609. doi: 10.3389/fcvm.2022.921609 (PMC9277029; doi:10.3389/fcvm.2022.921609)

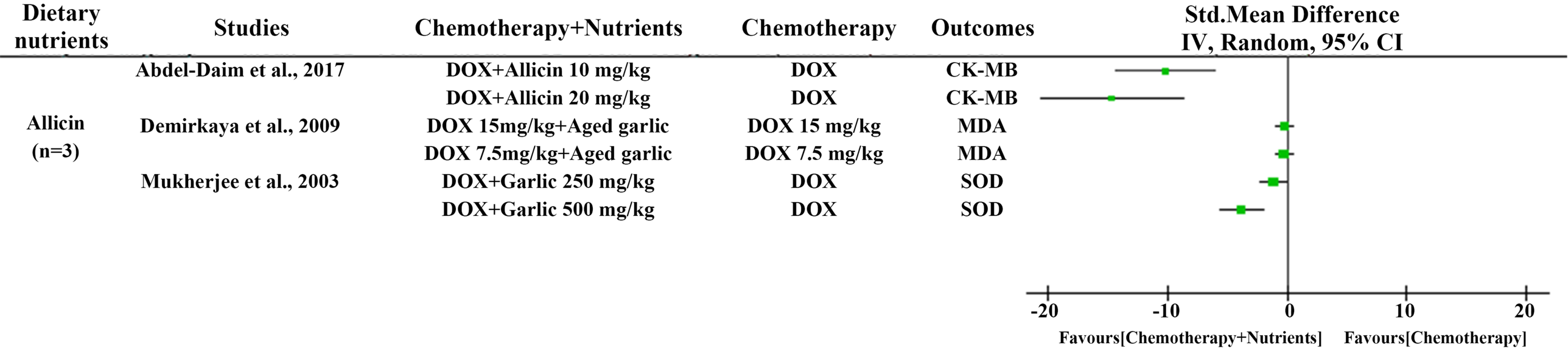

Supplement: Supplementary file 4 [file Image_2.TIF]

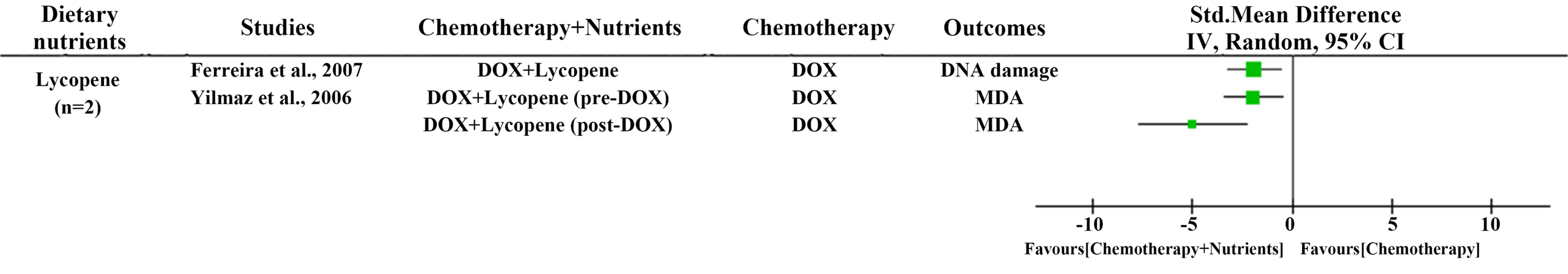

Supplement: Supplementary file 5 [file Image_3.TIF]

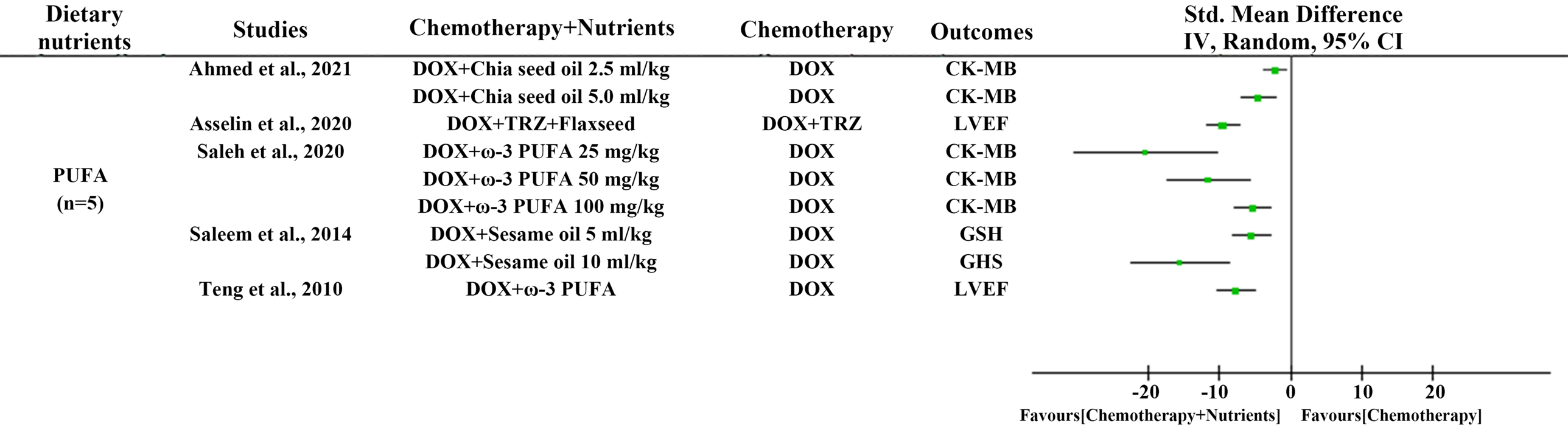

Supplement: Supplementary file 6 [file Image_4.TIF]

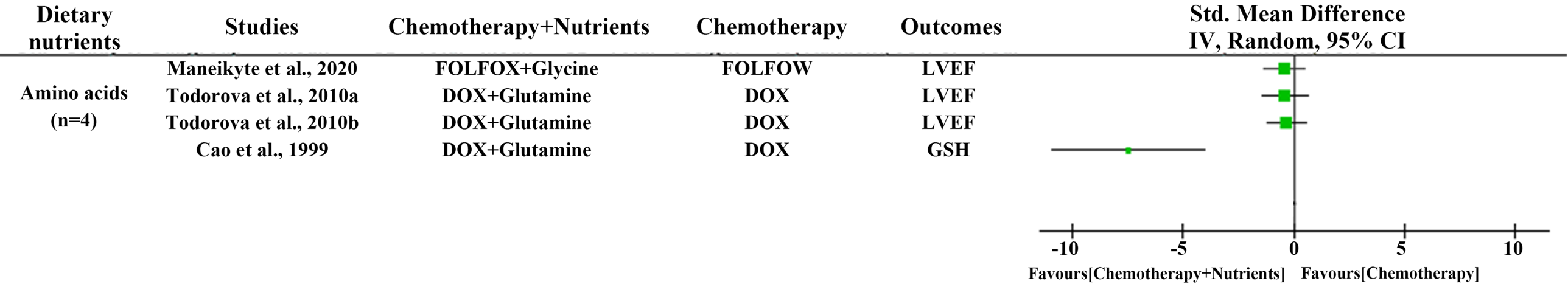

Supplement: Supplementary file 7 [file Image_5.TIF]

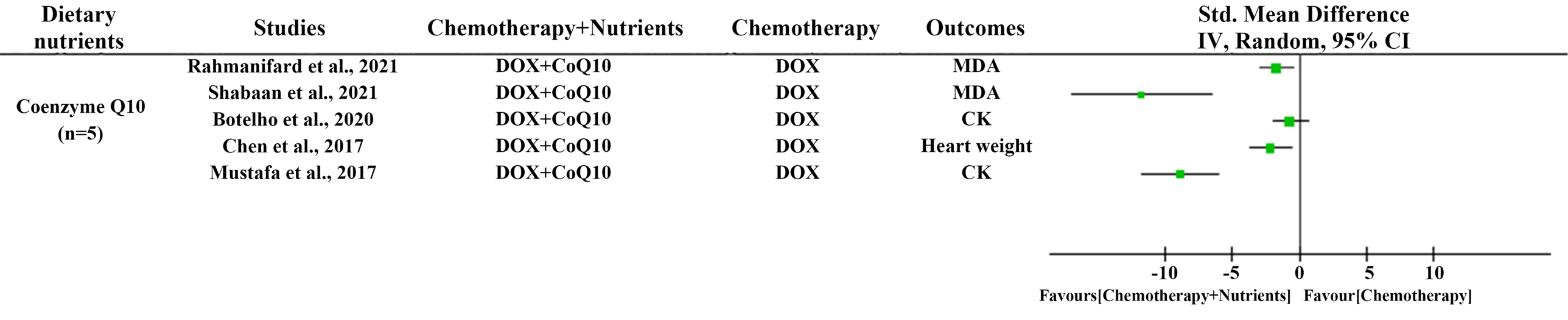

Supplement: Supplementary file 8 [file Image_6.TIF]

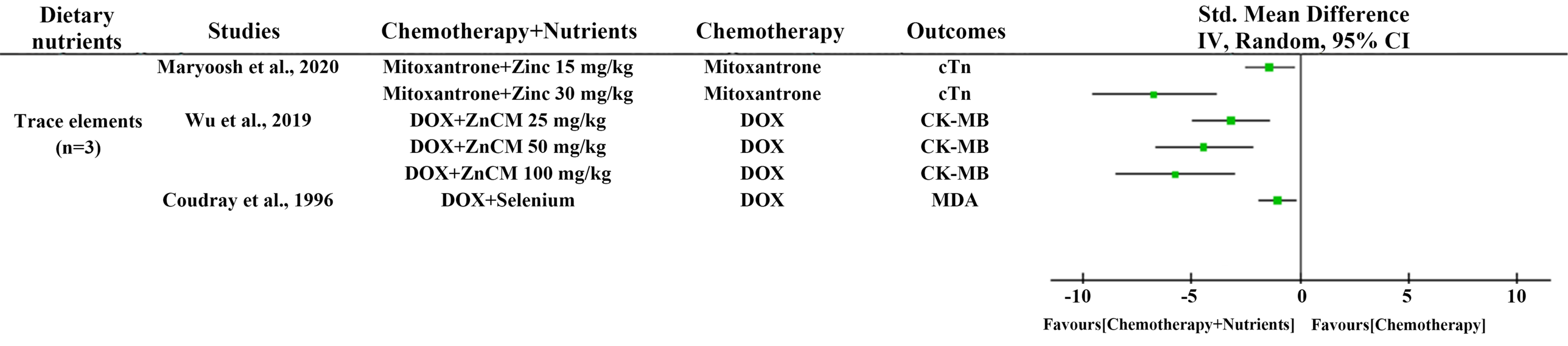

Supplement: Supplementary file 9 [file Image_7.TIF]

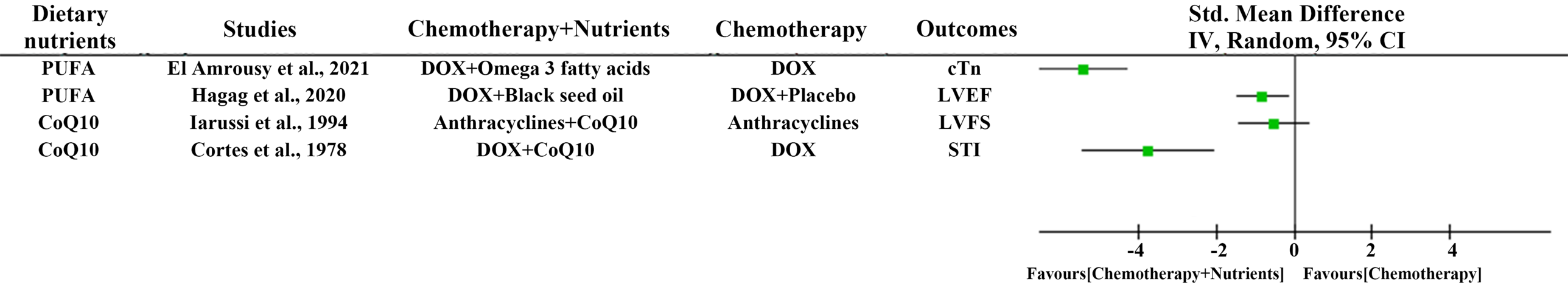

Supplement: Supplementary file 10 [file Image_8.TIF]
